# The Predictive Accuracy of Methods Commonly Used for Evaluating Animal Distress

**DOI:** 10.1096/fj.202504927RR

**Published:** 2026-06-08

**Authors:** Emily Leitner, Annika Thämlitz, Muhammad Imran Khan, Benjamin Schulz, Simone Kumstel, Brigitte Vollmar, Dietmar Zechner

**Affiliations:** ^1^ Rudolf‐Zenker‐Institute of Experimental Surgery Rostock University Medical Center Rostock Germany

**Keywords:** animal welfare, bile duct ligation, chronic pancreatitis, distress, receiver operating curves, refinement, robustness, transmitter implantation

## Abstract

Accurate distress assessment is essential for ensuring animal welfare and scientific validity. Although behavioral and physiological indicators are commonly used, their predictive value across disease models and severity grades remains insufficiently defined. Here, we evaluated the discriminatory performance of body weight changes, distress scoring, burrowing, and nesting across ten datasets involving transmitter implantation, bile duct ligation (BDL), and chronic pancreatitis (CP) in mice. Receiver operating characteristic analyses determined the area under the curve (AUC), and optimal cut‐offs derived from one dataset per model were applied to independent datasets to assess cross‐project accuracy. Under moderate to high distress conditions, such as BDL and the acute phase after transmitter implantation, distress score showed excellent discriminative performance (AUC ≥ 0.96), while body weight, burrowing, and nesting also performed fair to excellent (AUC ≥ 0.76). In contrast, during low‐distress conditions, including CP and recovery after implantation, discrimination was markedly reduced (body weight AUC ≥ 0.62; distress score, burrowing, and nesting AUC ≥ 0.50). When cut‐offs were applied to independent datasets, distress score (≥ 96%) and body weight (≥ 86%) maintained high accuracy under moderate to high distress, whereas burrowing and nesting performed less reliably (≥ 65%). Accuracy declined further under low distress with body weight ≥ 56% and distress score ≥ 50%. Behavioral measures showed poor accuracy (burrowing ≥ 39%, nesting ≥ 19%). Overall, burrowing and nesting can support distress evaluation under moderate to high distress, but are less reliable than body weight and distress score, particularly under low‐distress conditions.

## Graphical abstract

Predictive accuracy of body weight change, distress score, burrowing, and nesting behavior across multiple intervention phases.

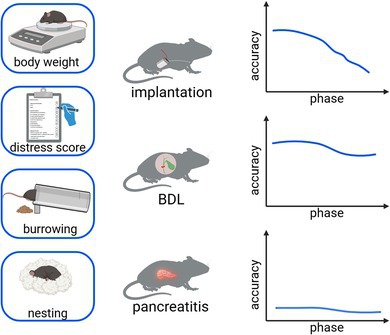

## 1 Introduction

Animal models remain essential for investigating complex physiological and pathophysiological processes, despite ongoing advances in alternative methods [1, 2]. However, animal experiments may compromise animal welfare by causing pain and distress [3, 4].

Extensive debates have led to the adoption of working definitions for pain, distress, and animal welfare. According to the revised definition of the International Association for the Study of Pain, pain is an unpleasant sensory and emotional experience associated with actual or potential tissue damage [5]. Because pain is a subjective experience, it can only be assessed indirectly in animals [5, 6, 7].

Distress is described as a negative state that arises when an animal is unable to adequately adapt to one or more stressors and can no longer cope successfully with its environment. It may result from disease processes, from pain, or experimental and husbandry procedures [6, 8]. Distress typically occurs when the biological costs of stress exceed the animal's adaptive capacity, resulting in disruptions of behavior, growth, reproduction, or immune function [6, 8]. Because distress, as a subjective negative state, cannot be measured directly, its assessment in laboratory animals relies on physiological and behavioral indicators whose relation to distress is indirect and context‐dependent [6, 8]. Animal welfare, in contrast, describes the overall state of an animal [6]. Pain and distress represent important negative components that can impair welfare [6], but welfare is influenced by a broader range of factors. A widely used framework for welfare assessment is the Five Domains Model, which integrates four physical or functional domains like nutrition, physical environment, health, and behavioral interactions, that together shape the fifth domain, the animal's mental state [9]. Although pain, distress, and animal welfare represent distinct concepts, experimental research often focuses on minimizing distress [10, 11, 12]. Pain may contribute to distress but represents only one of several potential causes, as distress can also arise from disease, environmental challenges, or experimental procedures [6, 13]. Consequently, assessing distress has been proposed as a more integrative approach to evaluating animal burden than focusing solely on pain [6, 14].

To guide ethical and responsible animal research, the principles of Replacement, Reduction, and Refinement (3Rs) have become increasingly important [15]. Within this framework, refinement plays a critical role in minimizing animal burden and improving experimental validity by optimizing handling, housing, and welfare monitoring [15, 16].

A key requirement of refinement is the accurate assessment of distress in laboratory animals [17]. Such an assessment ensures that any discomfort, pain, or stress can be identified early and mitigated effectively. Moreover, distress in laboratory animals can also be a potential scientific confounder, as stress can alter physiological and behavioral parameters and thereby influence experimental outcomes [18, 19]. Accordingly, reliable distress assessment is therefore essential to ensure both animal well‐being and the validity of scientific data. However, quantifying distress remains challenging, as it is affected by multiple biological and experimental factors, including, for example, species [13], strain [20, 21], and experimental model [10, 22]. These complexities underscore the need for determining which methods can define distress with high reliability within an animal model and across different experimental contexts and severities.

Several indicators are commonly used to assess animal distress in mice, including body weight [23, 24, 25], clinical or distress scores that combine multiple observation parameters such as appearance, posture, and activity [24, 26, 27], and behavioral measures such as burrowing and nesting activity, which reflect highly motivated, species‐typical activities that are sensitive to disturbances in well‐being and have therefore been proposed as early indicators of distress in rodents [12, 28, 29, 30]. Because these behaviors can be measured non‐invasively under standard housing conditions, they have been proposed as practical indicators of impaired well‐being in laboratory rodents.

Although these methods are widely implemented, their accuracy, defined as the combined sensitivity and specificity for detecting distress, may vary between animal models and experimental contexts. While some studies report that certain indicators, like burrowing and nest building, can be markers of distress [20], others highlight important limitations and variability in their performance [31, 32]. These discrepancies raise important questions about which indicators provide consistently reliable information about the level of distress experienced by laboratory animals.

Despite their frequent use in the assessment of animal distress, systematic evaluations of these indicators across multiple experiments, animal models, and distress phases remain scarce. In particular, it remains unclear how reliably these indicators perform across different levels of distress, whether their discriminatory capacity is consistent between projects, and whether cut‐off values derived in one study can be meaningfully applied to other studies. Addressing these gaps is essential for improving the reliability of distress assessment.

To fill this gap, the present study evaluates several widely used indicators of distress, including body weight change, distress score, burrowing activity, and nesting behavior [12, 23, 25, 26, 28] across three mouse models that differ substantially in their inherent severity of discomfort they induce. Transmitter implantation represents an acute surgical intervention associated with moderate distress during the immediate postoperative phase, followed by a recovery period in which distress typically declines [23, 33]. The second model, bile duct ligation (BDL), induces progressive cholestasis and liver inflammation, resulting in moderate to high distress without any apparent recovery [23, 34]. In contrast, chronic pancreatitis (CP) involves gradually developing inflammation [35], leading to comparatively mild, but long‐lasting distress [23, 36]. These distinct severity patterns provide a structured framework for investigating the reliability of distress indicators under conditions of moderate to high distress (during the acute phase after transmitter implantation and during all phases of cholestasis) and low distress (during CP and the middle and late recovery phase after transmitter implantation).

Using receiver operating characteristic (ROC) analyses, we assess how reliable each indicator distinguishes animals undergoing experimental interventions with expected distress from non‐distressed baseline conditions, how performance varies across different phases of each model, and whether cut‐off values obtained in one project can be applied to other projects within the same model. By comparing indicator performances across different distress conditions, this study provides an evidence‐based evaluation of the strengths and limitations of commonly used distress measures and identifies context‐dependent constraints that are critical for their effective applications.

## 2 Materials and Methods

Please note that additional figures and tables are provided in the Supporting Information (Figures S1–, S4, Tables S1–, S8).

### 2.1 Ethics Statement

All animal experiments were approved by the local ethics committee and public authority (Landesamt für Landwirtschaft, Lebensmittelsicherheit und Fischerei Mecklenburg‐Vorpommern) in accordance with the European Directive 2010/63/EU and national legislation. For all ethical approval file numbers of each study, see Table S1.

### 2.2 Data and Study Design

Data from ten independent mouse studies were compiled to evaluate the robustness and generalizability of behavioral and physiological indicators across different disease models. A detailed overview of included projects is provided in Table S1. The analyzed parameters included burrowing behavior, nesting behavior, body weight, and distress score. Data from these studies have been partially published (Table S1) but were reanalyzed for the present comparative approach.

### 2.3 Animals

Breeding pairs were originally purchased from Charles River Laboratories (Sulzfeld, Germany) and maintained at the animal facility of the University Medical Center Rostock under specific pathogen‐free (SPF) conditions. Mice were single‐housed in Eurostandard Type III cages (Zoonlab GmbH, Castrop‐Rauxel, Germany) under a 12 h light/dark cycle, at 21°C ± 2°C and a relative humidity of 60% ± 20%. Food (10 mm pellets; ssniff‐Spezialdiäten GmbH, Soest, Germany) and tap water were provided *ad libitum*. Environmental enrichment included shredded tissue paper (PZN03058052, FSMED Verbandmittel GmbH, Frankenberg, Germany), a paper tunnel (75 × 38 mm, H 0528–151, ssniff, Germany), and a wooden block (40 × 16 × 10 mm, H0234.NSG, Abedd, Vienna, Austria).

### 2.4 Experimental Models

#### 2.4.1 Transmitter Implantation

To assess the reproducibility of distress‐related readouts after transmitter implantation, data from two studies were analyzed [37, 38]. Ten male C57BL/6J mice (project 1 = P1) and ten male C57BL/6J mice (project 2 = P2) underwent intraperitoneal implantation of ETA‐F10 transmitters (Data Science International, St. Paul, MN, USA). The negative electrode was sutured to the *Musculus pectoralis major*, and the positive electrode to the *Musculus obliquus externus abdominis* [37, 38]. Analgesia was provided via metamizole (Ratiopharm, Ulm, Germany) in the drinking water at concentrations of 1.25 g/L (P1) or 3 g/L (P2). Data were collected during the preoperative (pre) phase, on day 0 (acute), day 1 (early), days 7–8 (middle), and days 13–14 (late) post‐surgery. Across all animal models, the acute phase was defined as the day of the first intervention, whereas the late phase corresponded to the final measurements obtained before tissue harvest. The middle phase was selected to be approximately equidistant between the early and late phases, and the early phase was chosen to occur shortly after the acute phase.

#### 2.4.2 Bile Duct Ligation

Three independent datasets describing distress after bile duct ligation (BDL) [34, 38, 39], comprised 50 male BALB/c (project 3 = P3), 10 male C57BL/6J (project 4 = P4), and 55 male BALB/c (project 5 = P5) mice. Following a midline laparotomy, the common bile duct was carefully isolated and ligated three times using 5–0 polyester sutures. In projects 3 and 5, the bile duct was additionally severed between the ligatures [34, 39]. Analgesia was provided via metamizole in the drinking water (3 g/L for P3‐4, 1.25 g/L for P5). Measurements were obtained during the preoperative, acute (day 0), early (day 1), middle (day 5 or 7), and late (day 12 or 13) phases.

#### 2.4.3 Chronic Pancreatitis

Chronic pancreatitis (CP) was induced by repeated intraperitoneal injections of cerulein (50 μg/kg; Bachem, Bubendorf, Switzerland) dissolved in 0.9% sodium chloride. Injections were administered three times per day, three days per week for four consecutive weeks (days 0–33) [36]. The following cohorts included 25 male C57BL/6J (project 6 = P6), 16 male C57BL/6J (project 7 = P7), 16 male BALB/c (project 8 = P8), 16 female C57BL/6J (project 9 = P9), and 16 female BALB/c mice (project 10 = P10). Analgesia was provided via metamizole in drinking water (1.25 g/L for project 6, 3 g/L for P7‐10). Data were collected during the pre‐phase, acute (day 0), early (day 2), middle (day 16), and late (day 30) phases. No acute data were available for P6.

### 2.5 Evaluation of Animal Distress

The distress score was developed from the score sheet of Morton and Griffiths [27] and Paster et al. [26], and has been previously published [24]. This score is based on observational parameters, including body weight loss, general condition (e.g., fur condition, posture, hydration), spontaneous behavior, flight behavior, and procedure‐specific criteria such as wound healing. Each parameter is assigned a score point reflecting severity, and the total score represents the cumulative level of impairment, with higher scores indicating greater distress. On intervention days, this score was assessed 30 min after cerulein injection or recovery from anesthesia. Burrowing behavior was analyzed according to Deacon et al. [29]. A tube filled with 200 g of food pellets (10 mm, ssniff‐Spezialdiäten GmbH) was placed in the home cage 2–3.5 h before the dark phase. The displaced amount was measured after 2 h (P1‐4, P6‐10) or 17 h (P5). Nesting behavior was scored following Deacon et al. [28]. Mice received a 5 × 5 cm pressed cotton nestlet (Zoonlab GmbH) 30–60 min before the dark phase, and nests were scored the following morning on a 1–6 scale, with a score of 6 representing a near‐perfect nest with more than 90% of the circumference higher than the animal's height. Body weight was measured 1 day after each intervention and on corresponding observation days to capture post‐interventional effects and maintain consistent timing across measurements.

### 2.6 Statistical Analysis

To assess the discriminatory ability of each method to distinguish pre‐ and post‐interventional states, receiver operating characteristic (ROC) curve analyses were performed separately for each project and phase using GraphPad Prism (v10.6.1, San Diego, CA, USA). For each ROC analysis, the area under the curve (AUC), the associated *p*‐values, and optimal cut‐off values (based on the maximum Youden's index) were determined. Based on previous publications, the discriminatory performance of each parameter was classified as excellent (AUC > 0.9), good (0.8–0.9), fair (0.7–0.8), poor (0.6–0.7), or failed (0.5–0.6) [40, 41].

Cut‐off values derived from P1 (transmitter implantation), P3 (BDL), and P7 (CP) were applied across all datasets within the respective animal model to classify animals as “non‐distressed” or “distressed” and to calculate sensitivity, specificity, and accuracy. The selection of these reference projects was based on data availability and completeness. P1 was chosen for P1 and P2 because these data were older and already published, P3 was used for P3‐5 due to larger sample sizes and more complete datasets, and P7 was applied to P6‐10, considering sex differences and missing acute‐phase data in P6. The optimal cut‐off was defined as the value that maximized Youden's index (defined as (sensitivity + specificity) –1) [42]. In cases where multiple candidate cut‐offs yielded the same maximum Youden's index, the positive likelihood ratio, defined as sensitivity/(1—specificity), was used as a secondary criterion, choosing the cut‐off showing the highest finite likelihood ratio [42, 43, 44]. Diagnostic performance of these cut‐offs was assessed by classifying measurements as true non‐distressed (TN, correctly identified as non‐distressed mice during baseline period), false distressed (FD, incorrectly identified as distressed during baseline period), true distressed (TD, correctly identified as distressed during distress phases after experimental interventions), or false non‐distressed (FN, incorrectly identified as non‐distressed during distress phases after experimental interventions). It should be noted that the threshold direction was parameter‐dependent: body weight, burrowing, and nesting were classified as distressed (i.e., TD, FD when falling below the cut‐off), whereas the distress score was classified as distressed when exceeding the cut‐off. Based on the calculations, the diagnostic metrics were calculated as follows:

$$\text{Sensitivity}=\frac{\mathrm{TD}}{\mathrm{TD}+\mathrm{FN}}$$

$$\text{Specificity}=\frac{\mathrm{TN}}{\mathrm{TN}+\mathrm{FD}}$$

$$\text{Accuracy}=\frac{\mathrm{TD}+\mathrm{TN}}{\mathrm{TD}+\mathrm{FN}+\mathrm{TN}+\mathrm{FD}}$$

To compare means across groups, GraphPad Prism v10.6.1 was used. Normality of data was assessed using the Shapiro–Wilk test. For normally distributed and repeated‐measures data, repeated‐measure ANOVA was applied. When datasets contained missing time points, mixed‐effects models were used. Non‐normally distributed repeated‐measures data were analyzed using the Friedman test with Dunn's post hoc correction. For independent group comparisons, one‐way ANOVA or Kruskal‐Wallis tests were used as appropriate, followed by Tukey's or Dunn's post hoc testing. Statistical significance was set at *p* ≤ 0.05.

Pairwise Spearman rank correlations (*ρ*) were computed between the four distress markers i.e., body weight change (BW), distress score (DS), burrowing activity (Burr), and nesting behavior (Nest). Correlations were calculated at the individual‐animal level, separately for each project (P1‐P10) and disease phase (pre, acute, early, middle, late), in R using the cor.test () function with method = “spearman” and exact = FALSE. Values were analyzed only when at least five complete pairs were available and both variables had non‐zero variance.

## 3 Results

To assess the ability of different parameters to distinguish non‐distressed mice (before any intervention) from animals at defined post‐intervention phases, ROC and corresponding AUCs were calculated to quantify discriminative performance (for examples see Figure S1).

### 3.1 Declining Discriminative Performance During Post‐Implantation Recovery

**FIGURE 1:**
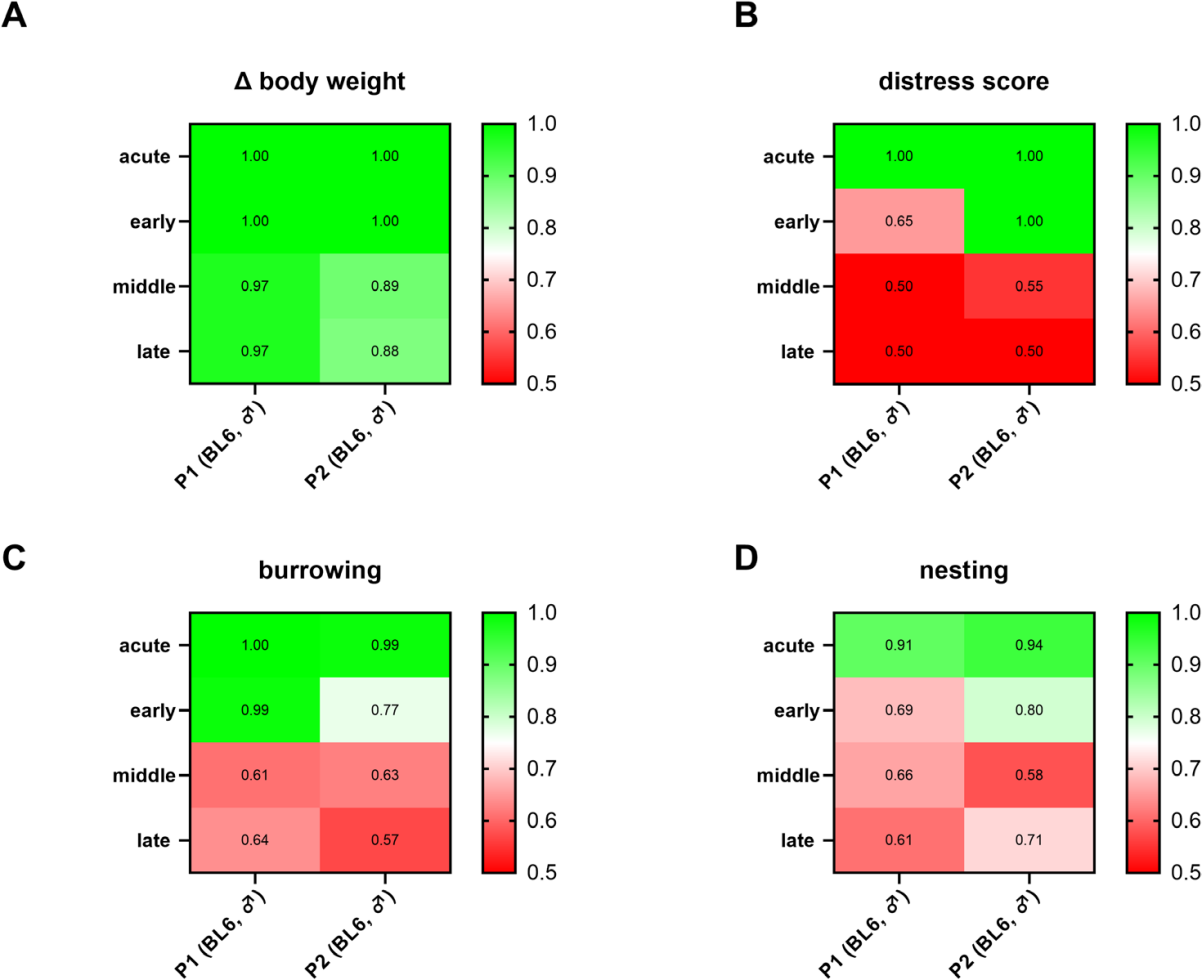
Discriminative performance of methods to differentiate mice before and across different phases after transmitter implantation. Shown are the area under the curve (AUC) values derived from receiver operating characteristic (ROC) analyses for (A) body weight changes, (B) distress score, (C) burrowing behavior, and (D) nesting behavior in two independent projects (P1 and P2). Higher AUC values indicate better discrimination between pre‐ and post‐intervention phases. Sample sizes: N (P1, BL6, ♂) = 10; n (P2, BL6, ♂) = 10.

After transmitter implantation, body weight consistently demonstrated good to excellent discriminative performance across all phases in two independent projects, with AUCs ≥ 0.88 (Figure 1A). Specifically, during the acute phase, the AUC reached 1.00, while in the middle or late phase it was ≥ 0.97 in P1 and ≥ 0.88 in P2 (Figure 1A). In contrast, distress score, burrowing, and nesting showed excellent discrimination only in the acute phases (AUC ≥ 0.91), while their performance declined with later phases (AUC 0.50–1.00) (Figure 1B–D).

**FIGURE 2:**
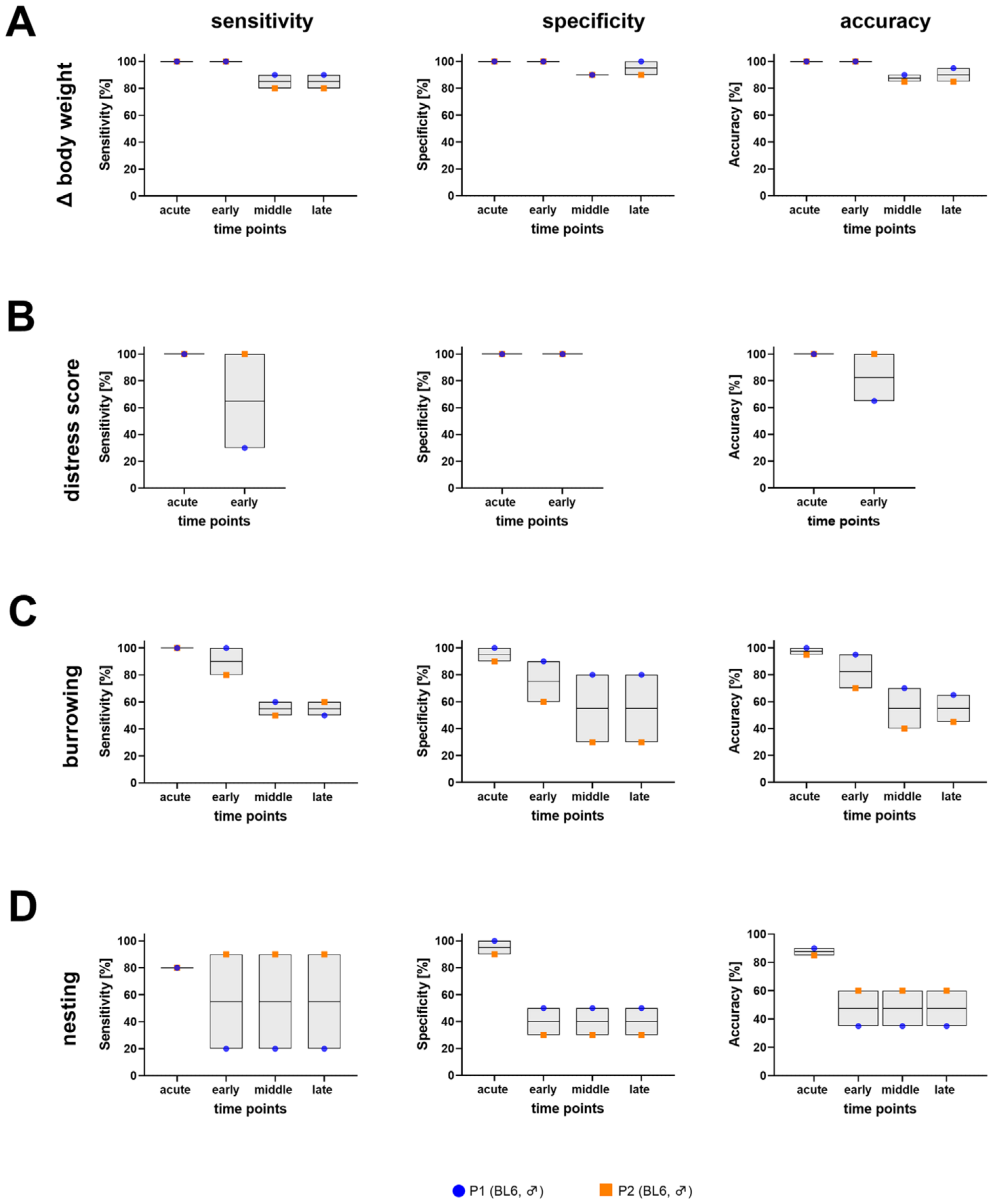
Performance of physiological and behavioral parameters across different phases after transmitter implantation. (A) Body weight changes, (B) distress score, (C) burrowing behavior, and (D) nesting behavior. Cut‐off values of P1 were applied to all data sets. Sample sizes: N (P1, BL6, ♂) = 10; n (P1, BL6, ♂) = 10. For the distress score, calculations for the middle and late phases were not possible because all animals exhibited a distress score of 0 in this phase. As a result, no contingency table could be generated and Youden's index as well as sensitivity, specificity, and accuracy could not be computed.

To identify optimal cut‐off values distinguishing non‐distressed animals from those with transmitter implants, Youden's index was calculated for each method and experimental phase in both projects. During the acute phase, the index was high across all parameters, with values consistently ≥ 0.8 (Table S2), but declined during later phases. Body‐weight cut‐offs derived from P1 yielded excellent classification across both datasets, with 100% sensitivity, specificity, and accuracy in the acute phase (Figure 2A). During middle and late phases, sensitivity remained ≥ 80%, specificity ≥ 90%, and accuracy ≥ 85%. Distress score cut‐offs similarly performed well acutely (100% accuracy), but accuracy dropped considerably during the early phase (65%–100%) (Figure 2B). Burrowing and nesting showed high acute‐phase accuracy (burrowing 95%–100%, nesting 85%–90%), but their performance declined markedly in middle and late phases (40%–70% for burrowing; 35%–60% for nesting Figure 2C,D).

Applying body‐weight cut‐offs from P1 led to consistent classifications across both datasets, with correct identification of truly distressed animals at 100% during acute and 80%–90% during the middle and late phases (discussed classification results are printed in bold in Table S3). Distress score cut‐offs showed perfect identification of truly distressed animals during the acute phase but increasingly variable results in the early phase (30%–100%). Burrowing and nesting cut‐offs also performed well during the acute phase (≥ 80%) but showed imperfect identification of truly distressed animals during the middle and late phases (50%–60% for burrowing and 20%–90% for nesting) (Table S3).

Overall, both discriminative power and cross‐project classification accuracy were highest during the acute period directly after transmitter implantation and decreased progressively during early, middle, and late recovery phases.

### 3.2 Robust Discriminative Performance by Body Weight and Distress Score After BDL

**FIGURE 3:**
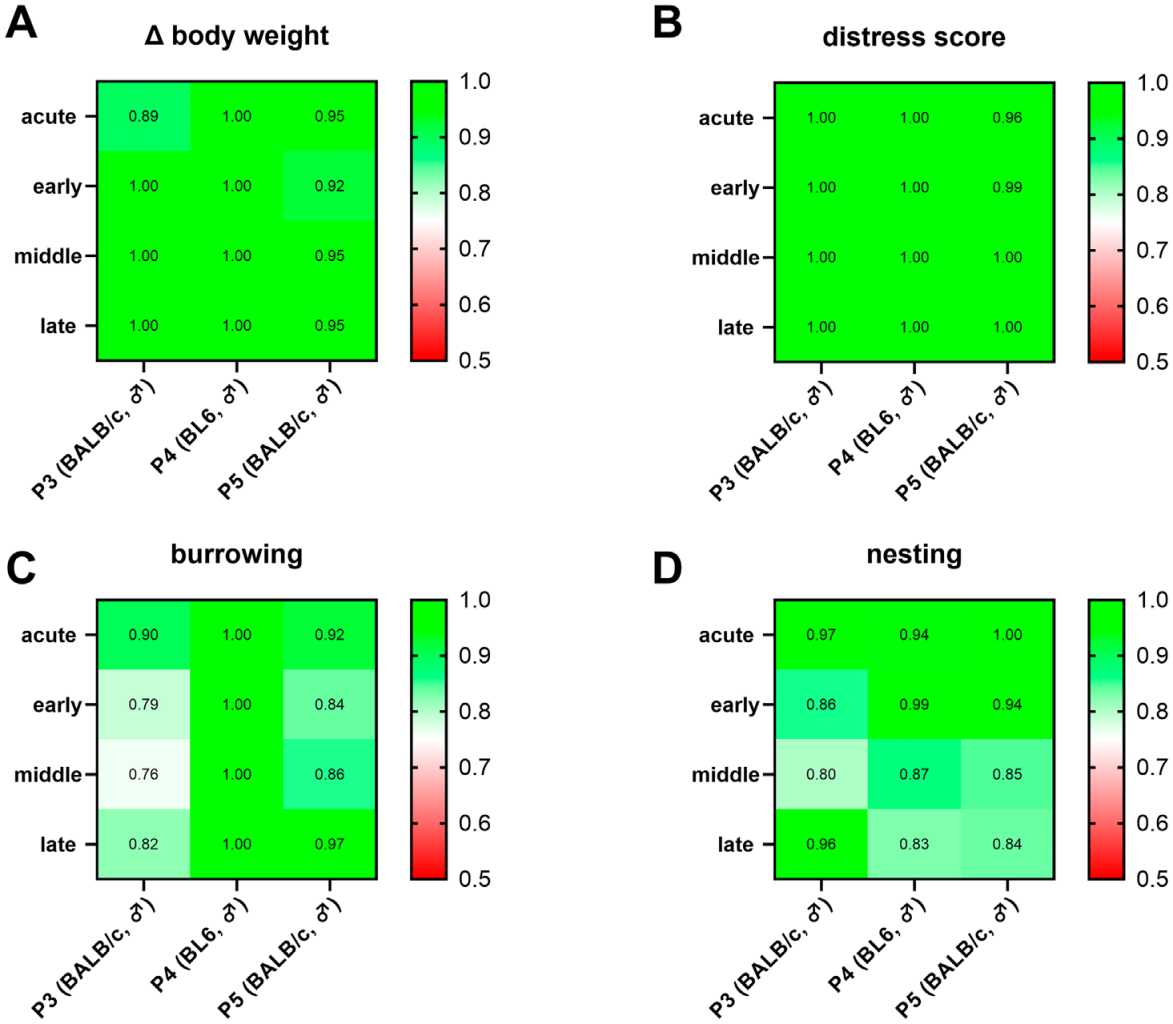
Discriminative performance of methods to differentiate mice before and across different phases after BDL. Shown are area under the curve (AUC) values derived from receiver operating characteristic (ROC) analyses for (A) body weight changes, (B) distress score, (C) burrowing behavior, and (D) nesting behavior in three independent projects (P3, P4, and P5). Higher AUC values indicate better discrimination between pre‐ and post‐intervention states. Sample sizes: N (P3, BALB/c, ♂) = 50; *n* (P4, BL6, ♂) = 10; *n* (P5, BALB/c, ♂) = 55 (panels A, B), 26 (panels C, D).

**FIGURE 4:**
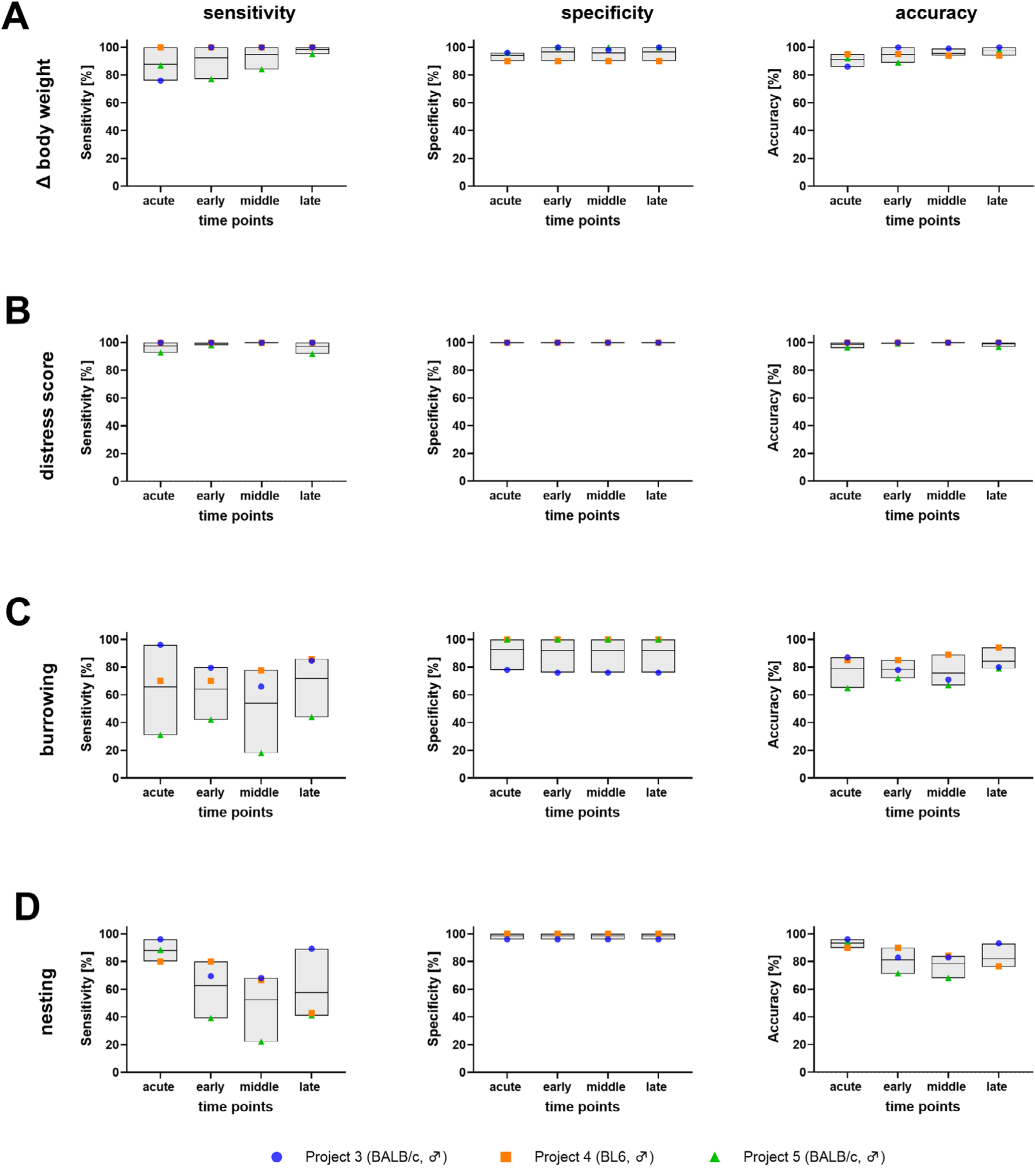
Performance of physiological and behavioral parameters across different phases after BDL. (A) Body weight changes, (B) distress score, (C) burrowing behavior, and (D) nesting behavior. Cut‐off values of P3 were applied to all data sets. Sample sizes: N (P3, BALB/c, ♂) = 50; *n* (P4, C57BL/6J, ♂) = 10; *n* (P5, BALB/c, ♂) = 55 (panels A, B), 24 (panels C, D).

Across three independent BDL projects (P3‐5), body weight and distress score consistently demonstrated good to excellent discriminative power (AUC ≥ 0.89, Figure 3A,B). Similarly, burrowing and nesting behaviors also demonstrated fair to excellent discriminative performance (AUC ≥ 0.76) across all experimental phases (Figure 3C,D). Youden's index was consistently high for body weight and distress score (≥ 0.7 in all phases), whereas burrowing and nesting showed broader variability (0.5–1.0, Table S4). Applying cut‐offs from P3 to all datasets resulted in high cross‐project accuracy for body weight (86%–100%) and distress score (96%–100%) (Figure 4A,B). In contrast, the behavioral parameters exhibited often lower accuracy with markedly greater variability. Burrowing accuracy ranged from 65% to 94%, while nesting accuracy varied between 68%–96% (Figure 4C,D). Correspondingly, body weight and distress score cut‐offs resulted in consistently high correct classification of true distressed states (76%–100%) across BDL datasets (Table S5). Behavioral cut‐offs were prone to misclassification, with correct true distressed rates ranging from 18%–96% for burrowing and 22%–96% for nesting (Table S5). Thus, across all phases after BDL, body weight and distress score showed the most robust discriminative performance and the lowest misclassification rates, whereas burrowing and nesting were less reliable.

### 3.3 Variable Discriminative Performance in CP Models

**FIGURE 5:**
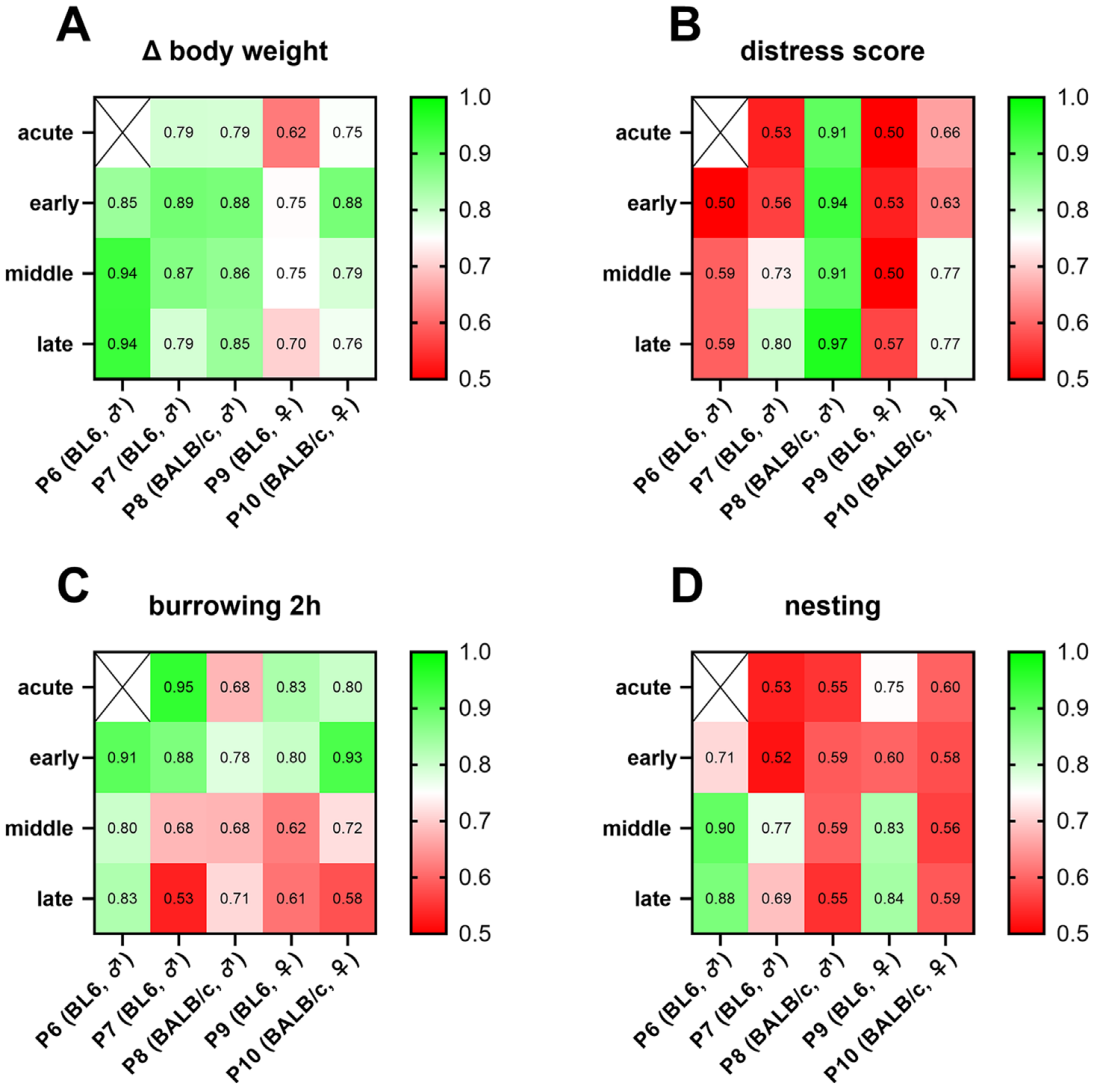
Discriminative performance of methods to differentiate mice before and across different phases during chronic pancreatitis. Shown are area under the curve (AUC) values derived from receiver operating characteristic (ROC) analyses for (A) body weight changes, (B) distress score, (C) burrowing behavior, and (D) nesting behavior in five independent projects (P6, P7, P8, P9, and P10). Higher AUC values indicate better discrimination between pre‐ and post‐intervention states. Sample sizes: *N* (P6, BL6, ♂) = 16, *n* (P7, BL6, ♂) = 16, *n* (P8, BALB/c, ♂) = 16, *n* (P9, BL6, ♀) = 16, *n* (P10, BALB/c, ♀) = 16.

In CP models, body weight showed fair to excellent discriminative ability in male mice (AUC ≥ 0.79), but performance was frequently lower in females (AUC 0.62–0.88) (Figure 5A). Distress score performed well only in P8 (BALB/c, male; AUC ≥ 0.91), but was substantially lower in all other projects (AUC 0.50–0.80) (Figure 5B). Burrowing behavior showed often fair to excellent discriminative power during the acute and early phases and good to excellent discriminatory ability in P6 (BL6, male) across all phases. In contrast, its discriminative performance declined during later phases in all other projects (Figure 5C). Nesting behavior generally ranged from failed to good discriminative power across all phases, with only P6 and P9 reaching good discriminative ability from the middle phase onwards (Figure 5D).

Youden's index values were generally ≤ 0.8, except for distress score in P8 (up to 0.9, Table S6) and cut‐offs differed markedly across projects and phases (Table S6).

**FIGURE 6:**
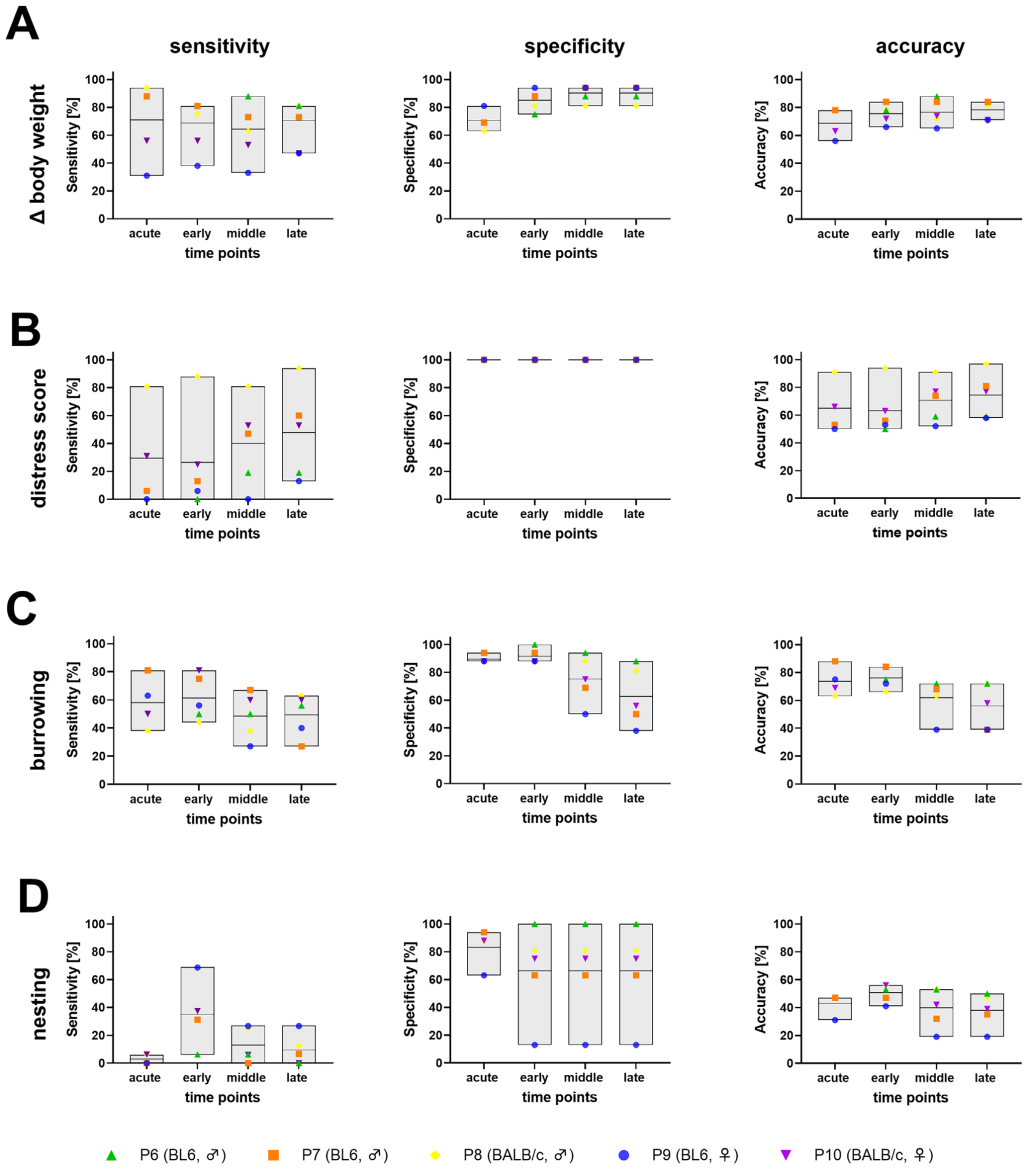
Performance of physiological and behavioral parameters across different phases during chronic pancreatitis. (A) Body weight changes, (B) distress score, (C) burrowing behavior, and (D) nesting behavior. Cut‐off values of P7 were applied to all data sets. Sample sizes: N (P6, BL6, ♂) = 16, n (P7, BL6, ♂) = 16, n (P8, BALB/c, ♂) = 16, n (P9, BL6, ♀) = 16, n (P10, BALB/c, ♀) = 16.

Applying the cut‐offs derived from P7 to all CP datasets revealed often low accuracy and substantial variability across projects. Body‐weight accuracy ranged from 56%–88% (Figure 6A). The distress score showed consistently high specificity (100%), but inconsistent accuracy (50%–97%, Figure 6B). Burrowing showed accuracies from 39%–88%, and nesting 19%–56% across projects (Figure 6C,D). Correspondingly, correct classification rates of true distressed animals varied widely (body weight 31%–94%, distress 0%–94%, burrowing 27%–81%, nesting 0%–69%; Table S7).

Thus, none of the methods used to assess distress in mice during CP provided robust discriminative performance. Cut‐offs derived from one dataset failed to generalize to others, resulting in low predictive performance and substantial between‐project variability.

### 3.4 Correlations Between Indicators of Distress

Correlations between the four distress indicators, body weight change, distress score, burrowing activity, and nesting behavior were highly heterogeneous and predominantly non‐significant when calculated for each project and phase (Figure S2). To address the limited statistical power within individual projects, we also computed correlations after pooling all animals within each disease model for each phase (Figure S3). Statistically significant correlations were mainly observed between distress score and body weight, as well as distress score and nesting behavior (Table S8). Statistically significant correlations were observed between distress score and body weight change for transmitter implantation in 1 out of 3 cases, after BDL in 3 out of 4 cases, and during CP in 3 out of 4 cases. Significant correlations were observed between distress score and nesting behavior for transmitter implantation in 1 out of 3 cases, after BDL in 2 out of 4 cases, and during CP in 2 out of 4 cases (Table S8). However, it should be noted that overall correlation coefficients were generally low and heterogeneous, irrespective of whether data were pooled within an animal model or analyzed separately (Figures S2, S3).

## 4 Discussion

### 4.1 The Discriminatory Power of Indicators Depends on the Distress Severity

In the present study, the term distress is used as an integrative concept describing a state of compromised well‐being resulting from an animal's reduced ability to cope with environmental challenges [6, 8, 9, 39]. This can arise from illness, pain, but also from other impairments or psychological stress [6, 8, 13, 45], and can be reflected by a range of behavioral and physiological alterations. Accordingly, the indicators evaluated here should not be interpreted as specific measures of a single component, such as pain or illness, but rather interpreted as indicators of distress.

The present study demonstrates that the discriminatory performance of common distress indicators varies substantially with the severity of distress experienced by laboratory mice, with the best performance occurring in situations with clearly elevated distress levels. This was most evident when comparing the acute postoperative phase following transmitter implantation to the middle and late recovery phases. Body weight change, the distress score, burrowing, and nesting behavior showed excellent discriminative ability, with AUC ≥ 0.91 when exploring the acute phase after transmitter implantation. Their performance, however, declined in the subsequent phases as the animals recovered (Figure 1). The premise that distress peaks immediately after transmitter implantation and then declines during postoperative recovery is well supported by previous studies [24, 33, 46]. Across these studies, parameters such as heart rate, locomotion, and fecal corticosterone metabolites followed the same temporal pattern, confirming a short, well‐defined peak of postoperative distress followed by a recovery within a few days [24, 33, 46].

A similar distinction emerged when comparing BDL with CP models. BDL induces sustained and pronounced distress, whereas CP is characterized by milder distress [23, 47]. Accordingly, AUCs demonstrated fair to excellent discriminatory power in BDL across all parameters (AUC ≥ 0.76, Figure 3) but were often lower and varied widely across the CP studies (AUC 0.50–0.97, Figure 5). Thus, this comparison also supports the conclusion that the discriminatory power of distress indicators is reduced in situations of subtle impairments in well‐being.

Overall, these findings demonstrate a fundamental principle. The discriminatory power of distress indicators increases with the magnitude of distress. Under conditions of pronounced distress, most indicators performed well. In contrast, during mild or inconsistent distress, such as in the CP model, classification performance was lower and became more variable between projects. This underscores the need for more sensitive assessment strategies for evaluating animal distress in low‐distress situations.

### 4.2 Accuracy of Cut‐Off Values Under Moderate to High‐ and Low‐ Distress Conditions

Cut‐off values derived using the Youden's index and validated across projects provided further insight into the performance of distress indicators under varying degrees of distress. Under moderate to high distress, such as the acute phase after transmitter implantation or all phases of BDL, cut off values derived from Youden's index classified animals with high accuracy, especially for body weight and distress score (≥ 86%), while burrowing and nesting maintained only moderate to good accuracy (≥ 65%) (Figure 2, Figure 4). This suggests that in moderate‐to‐high‐severity settings, both physiological and behavioral parameters can accurately indicate whether mice are experiencing distress. Among all measures, body weight and the distress score were the most reliable diagnostic indicators of distress. Misclassification rates were correspondingly low for body weight and distress score, as both correctly identified the majority of distressed animals (TD ≥ 76%), whereas behavioral measures showed substantially higher and more variable misclassification rates, reflecting their lower robustness under these conditions (TD ≥ 18%) (acute phases in Table S3 and all phases in Table S5).

In contrast, accuracy declined substantially during mild distress phases, such as the middle and late recovery phase after transmitter implantation and throughout CP. Even though body weight was the most reliable parameter, its accuracy was compared to the moderate‐to‐high distress situations often reduced, and its variability increased considerably (accuracy 56%–95% in middle and late phase in Figure 2 and all phases in Figure 6). The accuracy of the distress scoring (50%–97%) and especially behavioral readouts were also often low and performed inconsistently (19%–88% in middle and late phase in Figure 2 and all phases in Figure 6). The broad spread in true distressed rates (body weight 31%–94%, distress score 0%–94%, burrowing 27%–81%, nesting 0%–90%) highlights the poor robustness of cut‐off values under low‐severity conditions (middle and late phase in Table S3 and all phases in Table S7). Both the observed low accuracies and the inconsistent cross‐project accuracies clearly indicate that cut‐off values derived in mild‐distress settings are not reliably transferable to other projects and should, therefore, not be interpreted as universal thresholds. Thus, threshold‐based classification is reliable only when distress levels are moderate or high.

### 4.3 Lack of Robust Correlations Between Indicators of Distress

When evaluating correlations among changes in body weight, distress score, burrowing activity, and nesting behavior, three key observations emerge (Figure S2). First, correlations between these indicators of distress are generally low. Second, these correlations are not consistent across different projects. Third, in some cases, their direction is even reversed. For instance, the correlation between distress score and burrowing activity shown in Figure S2 is positive during the acute phase in P3, but negative in P4.

Several factors likely contribute to these findings. One issue is the lack of temporal alignment between measurements. For example, distress scores are typically assessed 30 min after surgical intervention, whereas burrowing and nesting behaviors are recorded later, during the night, and body weight is measured on the following day. Because these indicators reflect different time windows, they may capture distinct physiological and behavioral states, reducing the strength of correlations.

Additionally, not all measures are equally objective or continuous. Distress scoring and nesting assessments are subjective and are based on ordinal scales rather than true metric data. This may introduce variability and may reduce correlation coefficients.

Another important consideration is the presence of floor and ceiling effects, for example when assessing nesting and burrowing behavior. When animals cluster at the lower or upper bounds of these measures, variability is restricted, which in turn may attenuate correlations with other variables.

It is also important to consider that animals within a given phase may exhibit similarly high levels of distress, while individual differences remain small and somewhat random. As a result, correlations between distress indicators during a specific phase may be weak, even though the indicators can still effectively distinguish e.g., between pre‐ and post‐surgical states. This is illustrated by many low correlations observed, for example, during the acute phase in P1 and P2 (Figure S2), compared with the strong discriminatory performance demonstrated by high AUC values (≥ 0.91) in Figure 1.

Finally, genuine biological variability between animals may contribute to the observed heterogeneity. Individual differences in coping strategies, physiology, and behavior can lead to divergent responses across different indicators of distress.

Taken together, these low correlations among distress indicators also emphasize the need of integrating multiple methods within multivariate analytical frameworks when interpreting distress in animal studies.

### 4.4 Methodological Limitations

Although body weight measurement, distress scoring, burrowing activity assessment, and nesting behavior evaluation are established tools for assessing animal distress [12, 23, 24, 27, 29], their limitations must be carefully considered. For example, the distress score relies on observer‐based assessments, which can lead to substantial inter‐rater variability.

Burrowing behavior is limited by the narrow measurement range of the commonly used setup, which allows a maximum of only 200 g to be burrowed. This constraint can reduce sensitivity in detecting distress‐related decreases in burrowing, particularly when the selected burrowing duration is either too long or too short to capture meaningful differences. Therefore, careful selection of an appropriate assay duration is essential. Moreover, substantial baseline variability of burrowing activity between animals and across projects further complicates comparisons and may reduce the overall reliability of the assay see (Figure S4). Similarly, nesting behavior is influenced by the subjectivity inherent in scoring. In addition, considerable baseline variability across projects and between individual mice further limits comparability see (Figure S4).

These methodological limitations emphasize that while body weight and distress score serve as more stable indicators, behavioral parameters require careful standardization and contextual interpretation. In low distress models, more sensitive or multimodal indicators may be necessary to detect subtle changes in well‐being that are not adequately reflected by current behavioral assays. These methodological constraints likely contribute to the reduced classification accuracy observed during mild distress situations.

## 5 Conclusions

The present analysis demonstrates that the applicability and predictive accuracy of cut‐off values strongly depend on the underlying degree of distress. When distress is pronounced, physiological and behavioral changes caused by distress exceed biological variability, allowing classification. In contrast, under mild or fluctuating distress conditions, classification becomes unreliable due to low sensitivity or low specificity.

Across all three animal models and distress levels, body weight and the distress score showed higher discriminative performance and better accuracy than burrowing activity or nesting behavior. Thus, burrowing and nesting can support distress evaluation under moderate to high distress but are less reliable under low‐distress conditions. These findings also imply that fixed thresholds are suited for identifying pronounced distress but insufficient for capturing minor or gradual changes in animal well‐being. Future refinement efforts will therefore require more accurate diagnostic tools, especially for low distress situations.

## Author Contributions

D.Z. and B.V. conceived and designed the research; D.Z. supervised the project; D.Z. and B.V. administered the project and acquired funding; E.L. performed the research, acquired and curated the data, analyzed the data, and visualized the results; M.I.K. re‐examined all data and performed the analysis and visualization of all correlations. A.T., B.S., and S.K. performed the research and curated the data; all authors were involved in drafting and revising the manuscript and have read and agreed to the published version of the manuscript.

## Funding

This research was funded by the Deutsche Forschungsgemeinschaft DFG research group (FOR 2591, ZE 712/1–2, ZE 712/1–3, VO 450/15–2) and (VO 450/15–3).

## Disclosure

Institutional Review Board Statement: The animal experiments were approved by the local authority, Landesamt für Lebensmittelsicherheit und Fischerei Mecklenburg‐Vorpommern. All experiments were conducted in accordance with the German Animal Protection Law and the European Directive 2010/63/EU.

## Conflicts of Interest

The authors declare no conflicts of interest.

## Supporting information


**Figure S1:** ROC curves illustrate discriminatory power of body weight changes after transmitter implantation. Examples of ROC curves are shown for (A) the early and (B) the late phase after transmitter implantation, each compared to baseline values. *n* (P1) = 10. AUC represents the area under the curve and indicates discriminative ability.

**Figure S2:** Pairwise Spearman correlations between indicators of distress at each phase, shown separately for every individual project. Heatmap of Spearman correlation coefficients (*ρ*) between all pairwise combinations of body weight (BW), distress score (DS), burrowing (Burr) and nesting (Nest) computed within each project (P1‐P10) at every experimental phase (pre, acute, early, middle, late). Each row represents a pair (e.g., DS vs. Nest, BW vs. Burr), and each column corresponds to a phase within a given project. Cell color reflects the magnitude of coefficients, ranging from red (*ρ* = + 1, positive correlation) through white (*ρ* ≈0, no correlation) to blue (*ρ* = −1, negative correlation). Asterisks indicate statistically significant correlations (*p < 0.05, **p < 0.01, ***p < 0.001).

**Figure S3:** Disease‐model‐specific pairwise correlations between indicators of distress at each phase, after pooling animals within each model. Heatmap of Spearman correlation coefficients (*ρ*) between all pairwise combinations of body weight (BW), distress score (DS), burrowing (Burr) and nesting (Nest), computed at each phase (pre, acute, early, middle, late) after pooling all animals within each of the three disease model i.e., Transmitter implantation, bile duct ligation (BDL), and chronic pancreatitis (CP). Cell color reflects the magnitude of coefficients, ranging from red (*ρ* = + 1, positive correlation) through white (*ρ* ≈0, no correlation) to blue (*ρ* = −1, negative correlation). Asterisks indicate statistically significant correlations (*p < 0.05, **p < 0.01, ***p < 0.001, ns = not significant).

**Figure S4:** Comparison of distress parameters in the pre‐phase across all projects. Boxplots and Heat maps of (A,B) body weight changes (C,D) distress score (E,F) burrowing, and (G,H) nesting behavior across all projects. In panel A, body weight changes are shown relative to pre‐phase baseline measurements, panels C, E, and G show median values with individual data points, while panels B, D, F, and H display heat maps of corresponding *p*‐values. Statistical analysis was performed using the Kruskal‐Wallis test with Dunn's post hoc correction. Values of *p* < 0.05 were considered statistically significant. *n* (P1) = 10, *n* (P2) = 2, *n* (P3) = 50, *n* (P4) = 10, *n* (P5) = 55 (A‐D), 26 (E‐H), *n* (P6) = 16, *n* (P7) = 16, *n* (P8) = 16, *n* (P9) = 16, *n* (P10) = 16.

**Table S1:** Overview of mice used in each project.

**Table S2:** Cut‐Off values based on Youden‘s index for body weight change, distress score, burrowing, and nesting behavior after transmitter implantation in mice.

**Table S3:** Overview of classification results (true/false sick/healthy) for various distress parameters after transmitter implantation, when applying cut‐offs from P1 to P1 and P2.

**Table S4:** Cut‐Off values based on Youden‘s index for body weight change, distress score, burrowing, and nesting behavior after BDL.

**Table S5:** Overview of classification results (true/false sick/healthy) for various distress parameters after BDL, when applying the cut‐offs from P3 to P3, P4, and P5.

**Table S6:** Cut‐Off values based on Youden‘s index for body weight change, distress score, burrowing, and nesting behavior during pancreatitis.

**Table S7:** Overview of classification results (true/false sick/healthy) for various distress parameters during pancreatitis, when applying cut‐offs from P7 to P6, P7, P8, P9, and P10.

**Table S8:** Summary of Spearman correlations between all pairwise combinations of the four welfare markers (BW, DS, Burr, Nest) across the three disease models. For each model–pair combination, the total number of correlations tested (across projects and phases), the number reaching statistical significance, and the corresponding percentage are reported.

## Data Availability

Stored in repository.
