# Supplementary material for: The Predictive Accuracy of Methods Commonly Used for Evaluating Animal Distress
Source: FASEB J. 2026 Jun 8;40(11):e71986. doi: 10.1096/fj.202504927RR (PMC13244802; doi:10.1096/fj.202504927RR)
Supplement: Supplementary file 1 — Figure S1: ROC curves illustrate discriminatory power of body weight changes after transmitter implantation. Examples of ROC curves are shown for (A) the early and (B) the late phase after transmitter implantation, each compared to baseline values. n (P1) = 10. AUC represents the area under the curve and indicates discriminative ability. [file FSB2-40-e71986-s009.docx]

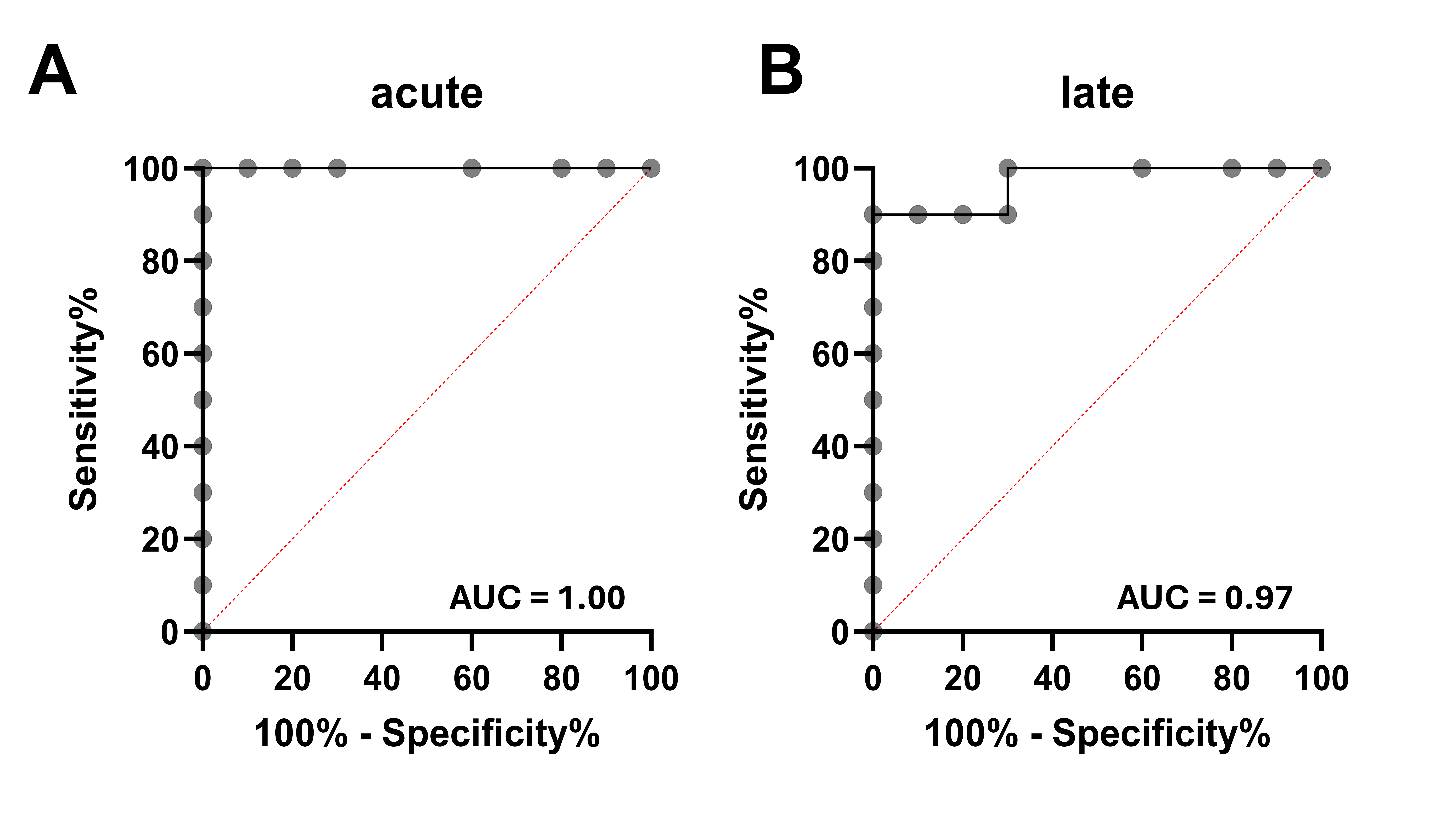


**Fig. S1: ROC curves illustrate discriminatory power of body weight changes after transmitter implantation.** Examples of ROC curves are shown for **(A)** the early and **(B)** the late phase after transmitter implantation, each compared to baseline values. n(P1) = 10. AUC represents the area under the curve and indicates discriminative ability
