# Supplementary material for: The Predictive Accuracy of Methods Commonly Used for Evaluating Animal Distress
Source: FASEB J. 2026 Jun 8;40(11):e71986. doi: 10.1096/fj.202504927RR (PMC13244802; doi:10.1096/fj.202504927RR)
Supplement: Supplementary file 2 — Figure S2: Pairwise Spearman correlations between indicators of distress at each phase, shown separately for every individual project. Heatmap of Spearman correlation coefficients (ρ) between all pairwise combinations of body weight (BW), distress score (DS), burrowing (Burr) and nesting (Nest) computed within each project (P1‐P10) at every experimental phase (pre, acute, early, middle, late). Each row represents a pair (e.g., DS vs. Nest, BW vs. Burr), and each column corresponds to a phase within a given project. Cell color reflects the magnitude of coefficients, ranging from red (ρ = + 1, positive correlation) through white (ρ ≈0, no correlation) to blue (ρ = −1, negative correlation). Asterisks indicate statistically significant correlations (*p < 0.05, **p < 0.01, ***p < 0.001). [file FSB2-40-e71986-s006.docx]

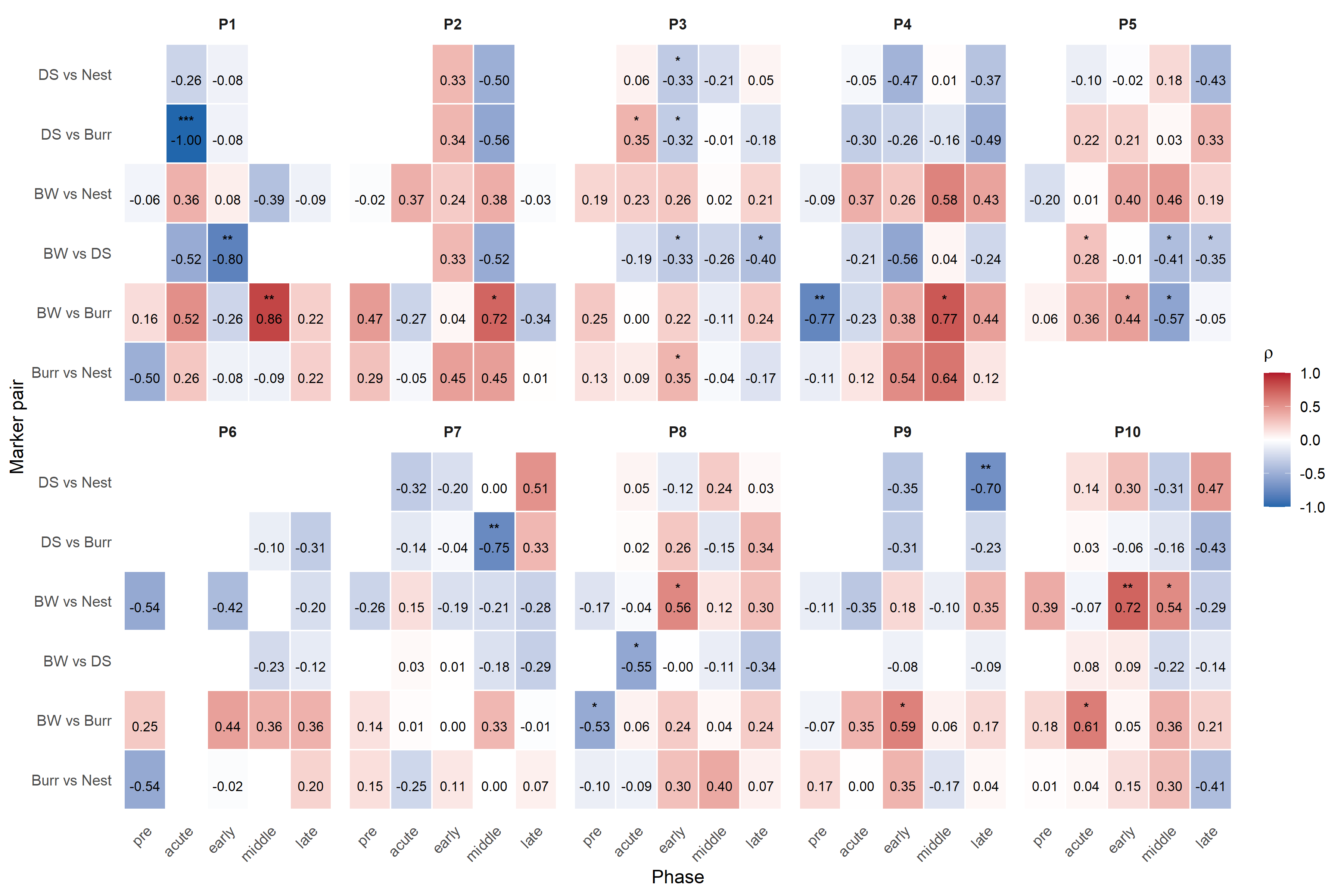


**Fig. S2: Pairwise Spearman correlations between indicators of distress at each phase, shown separately for every individual project.** Heatmap of Spearman correlation coefficients (ρ) between all pairwise combinations of body weight (BW), distress score (DS), burrowing (Burr) and nesting (Nest) computed within each project (P1-P10) at every experimental phase (pre, acute, early, middle, late). Each row represents a pair (e.g., DS vs Nest, BW vs Burr), and each column corresponds to a phase within a given project. Cell color reflects the magnitude of coefficients, ranging from red (ρ = +1, positive correlation) through white (ρ ≈ 0, no correlation) to blue (ρ = -1, negative correlation). Asterisks indicate statistically significant correlations (*p < 0.05, **p < 0.01, ***p < 0.001).
