# Supplementary material for: The Predictive Accuracy of Methods Commonly Used for Evaluating Animal Distress
Source: FASEB J. 2026 Jun 8;40(11):e71986. doi: 10.1096/fj.202504927RR (PMC13244802; doi:10.1096/fj.202504927RR)
Supplement: Supplementary file 3 — Figure S3: Disease‐model‐specific pairwise correlations between indicators of distress at each phase, after pooling animals within each model. Heatmap of Spearman correlation coefficients (ρ) between all pairwise combinations of body weight (BW), distress score (DS), burrowing (Burr) and nesting (Nest), computed at each phase (pre, acute, early, middle, late) after pooling all animals within each of the three disease model i.e., Transmitter implantation, bile duct ligation (BDL), and chronic pancreatitis (CP). Cell color reflects the magnitude of coefficients, ranging from red (ρ = + 1, positive correlation) through white (ρ ≈0, no correlation) to blue (ρ = −1, negative correlation). Asterisks indicate statistically significant correlations (*p < 0.05, **p < 0.01, ***p < 0.001, ns = not significant). [file FSB2-40-e71986-s005.docx]

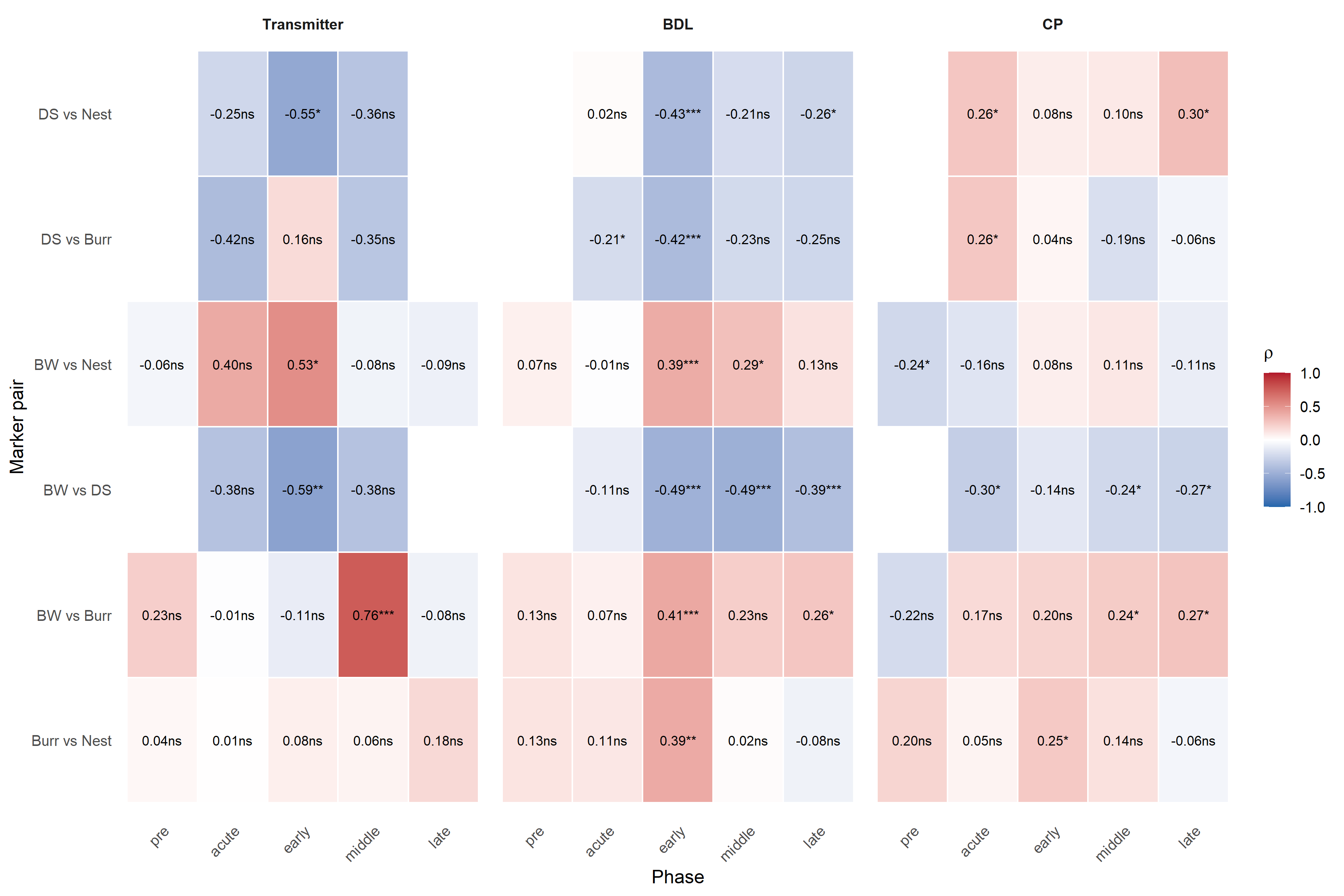


**Fig. S3:** **Disease-model-specific pairwise correlations between indicators of distress at each phase, after pooling animals within each model**. Heatmap of Spearman correlation coefficients (ρ) between all pairwise combinations of body weight (BW), distress score (DS), burrowing (Burr) and nesting (Nest), computed at each phase (pre, acute, early, middle, late) after pooling all animals within each of the three disease model i.e. Transmitter implantation, bile duct ligation (BDL), and chronic pancreatitis (CP). Cell color reflects the magnitude of coefficients, ranging from red (ρ = +1, positive correlation) through white (ρ ≈ 0, no correlation) to blue (ρ = -1, negative correlation). Asterisks indicate statistically significant correlations (*p < 0.05, **p < 0.01, ***p < 0.001, ns = not significant).
