# Supplementary material for: The Predictive Accuracy of Methods Commonly Used for Evaluating Animal Distress
Source: FASEB J. 2026 Jun 8;40(11):e71986. doi: 10.1096/fj.202504927RR (PMC13244802; doi:10.1096/fj.202504927RR)
Supplement: Supplementary file 4 — Figure S4: Comparison of distress parameters in the pre‐phase across all projects. Boxplots and Heat maps of (A,B) body weight changes (C,D) distress score (E,F) burrowing, and (G,H) nesting behavior across all projects. In panel A, body weight changes are shown relative to pre‐phase baseline measurements, panels C, E, and G show median values with individual data points, while panels B, D, F, and H display heat maps of corresponding p‐values. Statistical analysis was performed using the Kruskal‐Wallis test with Dunn's post hoc correction. Values of p < 0.05 were considered statistically significant. n (P1) = 10, n (P2) = 2, n (P3) = 50, n (P4) = 10, n (P5) = 55 (A‐D), 26 (E‐H), n (P6) = 16, n (P7) = 16, n (P8) = 16, n (P9) = 16, n (P10) = 16. [file FSB2-40-e71986-s008.docx]

**
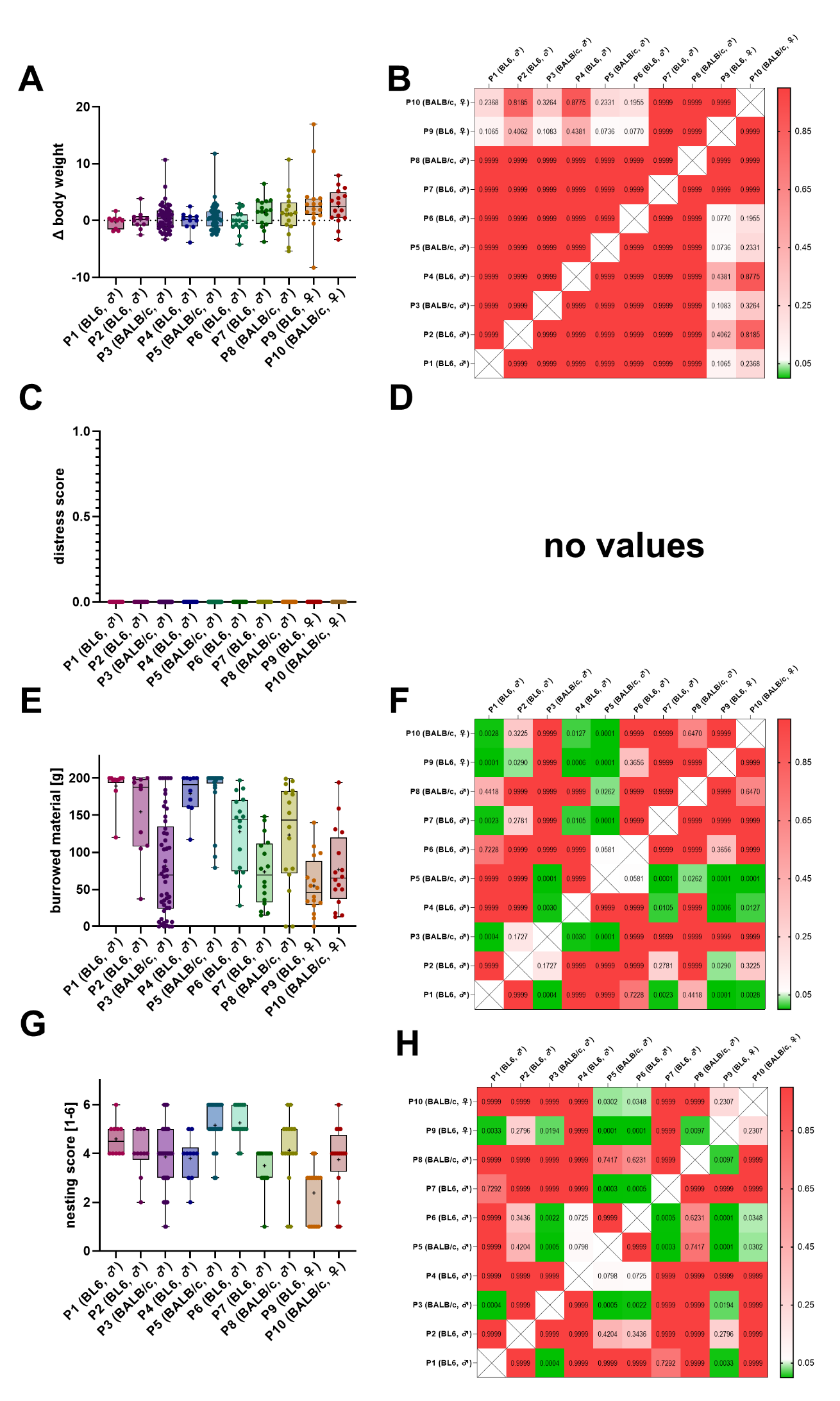
**

**Fig. S4: Comparison of distress parameters in the pre-phase across all projects.** Boxplots and Heat maps of **(A,B)** body weight changes, **(C,D)** distress score, **(E,F)** burrowing, and **(G,H)** nesting behavior across all projects. In panel A, body weight changes are shown relative to pre-phase baseline measurements, panels C, E, and G show median values with individual data points, while panels B, D, F, and H display heat maps of corresponding p-values. Statistical analysis was performed using the Kruskal-Wallis test with Dunn’s post hoc correction. Values of P<0.05 were considered statistically significant. n(P1)=10, n(P2)=2, n(P3)=50, n(P4)=10, n(P5)=55 (A-D), 26 (E-H), n(P6)=16, n(P7)=16, n(P8)=16, n(P9)=16, n(P10)=16.
