# Supplementary material for: The Predictive Accuracy of Methods Commonly Used for Evaluating Animal Distress
Source: FASEB J. 2026 Jun 8;40(11):e71986. doi: 10.1096/fj.202504927RR (PMC13244802; doi:10.1096/fj.202504927RR)
Supplement: Supplementary file 5 — Table S1: Overview of mice used in each project. [file FSB2-40-e71986-s001.docx]

**Table S1:** Overview of mice used in each project

| **project** | **mouse strain** | **sex** | **n** | **disease model / treatment groups** | **ethical approval file number** | **pubmed ID** | **abbreviation** |
| --- | --- | --- | --- | --- | --- | --- | --- |
| 1 | C57BL/6J | male | 10 | Transmitter implantation | -1-062/16 | 31641536 | P1 (BL6, ♂) |
| 2 | C57BL/6J | male | 10 | Transmitter implantation | -1-035/20 | 41463047 | P2 (BL6, ♂) |
| 3 | BALB/c | male | n(GSK805)=27  n(DMSO)=23 | Bile duct ligation | -1-035/20 | 39157975 | P3 (BALB/c, ♂) |
| 4 | C57BL/6J | male | 10 | Bile duct ligation | -1-035/20 | 41463047 | P4 (BL6, ♂) |
| 5 | BALB/c | male | n(MCC950)=29  n(control)=26 | Bile duct ligation | -1-022/17 | 31825086 | P5 (BALB/c, ♂) |
| 6 | C57BL/6J | male | n(miR 21 inhibitor)=12 n(miR 21 control)=13 | Pancreatitis | -1-022/17 | 31575986 | P6 (BL6, ♂) |
|  |  |  |  |  |  |  |  |
| 7 | C57BL/6J | male | n(GSK805)=8  n(DMSO)=8 | Pancreatitis | -1-070/20 | not published | P7 (BL6, ♂) |
| 8 | BALB/c | male | n(GSK805)=8  n(DMSO)=8 | Pancreatitis | -1-070/20 | not published | P8 (BALB/c, ♂) |
| 9 | C57/BL6J | female | n(GSK805)=8  n(DMSO)=8 | Pancreatitis | -1-070/20 | not published | P9 (BL6, ♀) |
| 10 | BALB/c | female | n(GSK805)=8  n(DMSO)=8 | Pancreatitis | -1-070/20 | not published | P10 (BALB/c, ♀) |
|  | | | |  |  |  |  |
