# Supplementary material for: The Predictive Accuracy of Methods Commonly Used for Evaluating Animal Distress
Source: FASEB J. 2026 Jun 8;40(11):e71986. doi: 10.1096/fj.202504927RR (PMC13244802; doi:10.1096/fj.202504927RR)
Supplement: Supplementary file 6 — Table S2: Cut‐Off values based on Youden‘s index for body weight change, distress score, burrowing, and nesting behavior after transmitter implantation in mice. [file FSB2-40-e71986-s004.docx]

**Table S2:** Cut-Off values based on Youden‘s index for body weight change, distress score, burrowing, and nesting behavior after transmitter implantation in mice.

| **parameter** | **phases** | **P1 (BL6, ♂)** | | **P2 (BL6, ♂)** | |
| --- | --- | --- | --- | --- | --- |
|  |  | **Youdens Index** | **Cut Off** | **Youdens Index** | **Cut Off** |
| **Δ body weight** | **pre vs. acute phase** | 1.0 | -3.8 | 1.0 | -4.4 |
|  | **pre vs. early phase** | 1.0 | -3.8 | 1.0 | -4.7 |
|  | **pre vs. middle phase** | 0.8 | -1.8 | 0.7 | -1.9 |
|  | **pre vs. late phase** | 0.9 | -1.9 | 0.7 | -2.0 |
| **distress score** | **pre vs. acute phase** | 1.0 | 2.5 | 1.0 | 3.5 |
|  | **pre vs. early phase** | 0.3 | 1.0 | 1 | 1 |
|  | **pre vs. middle phase** | no cut off values | no cut off values | 0.1 | 1 |
|  | **pre vs. late phase** | no cut off values | no cut off values | no cut off values | no cut off values |
| **burrowing** | **pre vs. acute phase** | 1.0 | 80.0 | 0.9 | 77 |
|  | **pre vs. early phase** | 0.9 | 168.5 | 0.6 | 104.5 |
|  | **pre vs. middle phase** | 0.4 | 195.0 | 0.3 | 199.0 |
|  | **pre vs. late phase** | 0.3 | 196.5 | 0.3 | 135.0 |
| **nesting** | **pre vs. acute phase** | 0.8 | 3.0 | 0.8 | 1.5 |
|  | **pre vs. early phase** | 0.3 | 4.5 | 0.5 | 3.5 |
|  | **pre vs. middle phase** | 0.3 | 4.5 | 0.2 | 4.5 |
|  | **pre vs. late phase** | 0.3 | 4.5 | 0.4 | 3.5 |
