# Supplementary material for: The Predictive Accuracy of Methods Commonly Used for Evaluating Animal Distress
Source: FASEB J. 2026 Jun 8;40(11):e71986. doi: 10.1096/fj.202504927RR (PMC13244802; doi:10.1096/fj.202504927RR)
Supplement: Supplementary file 7 — Table S3: Overview of classification results (true/false sick/healthy) for various distress parameters after transmitter implantation, when applying cut‐offs from P1 to P1 and P2. [file FSB2-40-e71986-s002.docx]

**Table S3:** Overview of classification results (true/false sick/healthy) for various distress parameters after transmitter implantation, when applying cut-offs from P1 to P1 and P2.

| **parameter** | **project** | **status** | **acute phase** | **early phase** | **middle phase [%]** | **late phase** |
| --- | --- | --- | --- | --- | --- | --- |
|  |  |  | **[%]** | **[%]** |  | **[%]** |
| **Δ body weight** | **P 1  (BL6, ♂)** | **TD** | **100** | 100 | **90** | **90** |
|  |  | **FN** | 0 | 0 | 10 | 10 |
|  |  | **FD** | 0 | 0 | 10 | 0 |
|  |  | **TN** | 100 | 100 | 90 | 100 |
|  | **P2 (BL6, ♂)** | **TD** | **100** | 100 | **80** | **80** |
|  |  | **FN** | 0 | 0 | 20 | 20 |
|  |  | **FD** | 0 | 0 | 10 | 10 |
|  |  | **TN** | 100 | 100 | 90 | 90 |
| **distress score** | **P 1  (BL6, ♂)** | **TD** | **100** | **30** | no cut off | no cut off |
|  |  | **FN** | 0 | 70 | no cut off | no cut off |
|  |  | **FD** | 0 | 0 | no cut off | no cut off |
|  |  | **TN** | 100 | 100 | no cut off | no cut off |
|  | **P2 (BL6, ♂)** | **TD** | **100** | **100** | no cut off | no cut off |
|  |  | **FN** | 0 | 0 | no cut off | no cut off |
|  |  | **FD** | 0 | 0 | no cut off | no cut off |
|  |  | **TN** | 100 | 100 | no cut off | no cut off |
| **burrowing** | **P1  (BL6, ♂)** | **TD** | **100** | 100 | **60** | **50** |
|  |  | **FN** | 0 | 0 | 40 | 50 |
|  |  | **FD** | 0 | 10 | 20 | 20 |
|  |  | TN | 100 | 90 | 80 | 80 |
|  | **P2 (BL6, ♂)** | **TD** | **100** | 80 | **50** | **60** |
|  |  | **FN** | 0 | 20 | 50 | 40 |
|  |  | **FD** | 10 | 40 | 70 | 70 |
|  |  | **TN** | 90 | 60 | 30 | 30 |
| **nesting** | **P1  (BL6, ♂)** | **TD** | **80** | 20 | **20** | **20** |
|  |  | **FN** | 20 | 80 | 80 | 80 |
|  |  | **FD** | 0 | 50 | 50 | 50 |
|  |  | **TN** | 100 | 50 | 50 | 50 |
|  | **P2**  **(BL6, ♂)** | **TD** | **80** | 90 | **90** | **90** |
|  |  | **FN** | 20 | 10 | 10 | 10 |
|  |  | **FD** | 10 | 70 | 70 | 70 |
|  |  | **TN** | 90 | 30 | 30 | 30 |

True distressed (TD), false non-distressed (FN), false distressed (FD), True non-distressed (TN)
