# Supplementary material for: The Predictive Accuracy of Methods Commonly Used for Evaluating Animal Distress
Source: FASEB J. 2026 Jun 8;40(11):e71986. doi: 10.1096/fj.202504927RR (PMC13244802; doi:10.1096/fj.202504927RR)
Supplement: Supplementary file 8 — Table S4: Cut‐Off values based on Youden‘s index for body weight change, distress score, burrowing, and nesting behavior after BDL. [file FSB2-40-e71986-s012.docx]

**Table S4:** Cu**t**-Off values based on Youden‘s index for body weight change, distress score, burrowing, and nesting behavior after BDL.

| **parameters** | **phases** | **P3 (BALB/c, ♂)** | | **P4 (BL6, ♂)** | | **P5 (BALB/c, ♂)** | |
| --- | --- | --- | --- | --- | --- | --- | --- |
|  |  | **Youden Index** | **Cut Off** | **Youden Index** | **Cut Off** | **Youden Index** | **Cut Off** |
| **Δ body weight** | **pre vs. acute phase** | 0.7 | -2.4 | 1.0 | -4.4 | 0.9 | -2.7 |
|  | **pre vs. early phase** | 1.0 | -3.6 | 1.0 | -8.1 | 0.9 | -2.6 |
|  | **pre vs. middle phase** | 1.0 | -2.9 | 1.0 | -7.0 | 0.9 | -2.6 |
|  | **pre vs. late phase** | 1.0 | -3.8 | 1.0 | -7.7 | 0.9 | -4.7 |
| **distress score** | **pre vs. acute phase** | 1.0 | 1.0 | 1.0 | 2.5 | 0.9 | 1.0 |
|  | **pre vs. early phase** | 1.0 | 1.0 | 1.0 | 2.0 | 1.0 | 1.0 |
|  | **pre vs. middle phase** | 1.0 | 1.0 | 1.0 | 3.5 | 1.0 | 1.0 |
|  | **pre vs. late phase** | 1.0 | 2.5 | 1.0 | 2.5 | 1.0 | 1.0 |
| **burrowing** | **pre vs. acute phase** | 0.8 | 19.0 | 1.0 | 86.5 | 0.7 | 172.5 |
|  | **pre vs. early phase** | 0.6 | 24.5 | 1.0 | 78.5 | 0.5 | 70.0 |
|  | **pre vs. middle phase** | 0.5 | 23.5 | 1.0 | 84.0 | 0.6 | 176.5 |
|  | **pre vs. late phase** | 0.7 | 23.0 | 1.0 | 75.0 | 0.9 | 177.0 |
| **nesting** | **pre vs. acute phase** | 0.9 | 1.5 | 0.8 | 2.5 | 0.9 | 3.5 |
|  | **pre vs. early phase** | 0.7 | 1.5 | 0.9 | 2.5 | 0.8 | 4.5 |
|  | **pre vs. middle phase** | 0.6 | 1.5 | 0.7 | 2.5 | 0.6 | 4.5 |
|  | **pre vs. late phase** | 0.9 | 1.5 | 0.6 | 2.5 | 0.5 | 4.5 |
