# Supplementary material for: The Predictive Accuracy of Methods Commonly Used for Evaluating Animal Distress
Source: FASEB J. 2026 Jun 8;40(11):e71986. doi: 10.1096/fj.202504927RR (PMC13244802; doi:10.1096/fj.202504927RR)
Supplement: Supplementary file 9 — Table S5: Overview of classification results (true/false sick/healthy) for various distress parameters after BDL, when applying the cut‐offs from P3 to P3, P4, and P5. [file FSB2-40-e71986-s010.docx]

**Table S5:** Overview of classification results (true/false sick/healthy) for various distress parameters after BDL, when applying the cut-offs from P3 to P3, P4, and P5.

| **parameter** | **project** | **status** | **acute phase** | **early phase** | **middle phase [%]** | **late phase** |
| --- | --- | --- | --- | --- | --- | --- |
|  |  |  | **[%]** | **[%]** |  | **[%]** |
| **Δ body weight** | **P3 (BALB/c, ♂)** | **TD** | **76** | **100** | **100** | **100** |
|  |  | **FN** | 24 | 0 | 0 | 0 |
|  |  | **FD** | 6 | 0 | 2 | 0 |
|  |  | **TN** | 94 | 100 | 98 | 100 |
|  | **P4 (BL6, ♂)** | **TD** | **100** | **100** | **100** | **100** |
|  |  | **FN** | 0 | 0 | 0 | 0 |
|  |  | **FD** | 10 | 10 | 10 | 10 |
|  |  | **TN** | 90 | 90 | 90 | 90 |
|  | **P5 (BALB/c, ♂)** | **TD** | **87** | **77** | **84** | **95** |
|  |  | **FN** | 13 | 23 | 16 | 5 |
|  |  | **FD** | 4 | 0 | 0 | 0 |
|  |  | **TN** | 96 | 100 | 100 | 100 |
| **distress score** | **P3 (BALB/c, ♂)** | **TD** | **100** | **100** | **100** | **100** |
|  |  | **FN** | 0 | 0 | 0 | 0 |
|  |  | **FD** | 0 | 0 | 0 | 0 |
|  |  | **TN** | 100 | 100 | 100 | 100 |
|  | **P4 (BL6, ♂)** | **TD** | **100** | **100** | **100** | **100** |
|  |  | **FN** | 0 | 0 | 0 | 0 |
|  |  | **FD** | 0 | 0 | 0 | 0 |
|  |  | **TN** | 100 | 100 | 100 | 100 |
|  | **P5 (BALB/c, ♂)** | **TD** | **93** | **98** | **100** | **92** |
|  |  | **FN** | 7 | 2 | 0 | 8 |
|  |  | **FD** | 0 | 0 | 0 | 0 |
|  |  | **TN** | 100 | 100 | 100 | 100 |
| **burrowing** | **P3 (BALB/c, ♂)** | **TD** | **96** | **80** | **66** | **85** |
|  |  | **FN** | 4 | 20 | 34 | 15 |
|  |  | **FD** | 22 | 24 | 24 | 24 |
|  |  | **TN** | 78 | 76 | 76 | 76 |
|  | **P4 (BL6, ♂)** | **TD** | **70** | **70** | **78** | **86** |
|  |  | **FN** | 30 | 30 | 22 | 14 |
|  |  | **FD** | 0 | 0 | 0 | 0 |
|  |  | **TN** | 100 | 100 | 100 | 100 |
|  | **P5 (BALB/c, ♂)** | **TD** | **31** | **42** | **18** | **44** |
|  |  | **FN** | 69 | 58 | 82 | 56 |
|  |  | **FD** | 0 | 0 | 0 | 0 |
|  |  | **TN** | 100 | 100 | 100 | 100 |
| **nesting** | **P3 (BALB/c, ♂)** | **TD** | **96** | **69** | **68** | **89** |
|  |  | **FN** | 4 | 31 | 32 | 11 |
|  |  | **FD** | 4 | 4 | 4 | 4 |
|  |  | **TN** | 96 | 96 | 96 | 96 |
|  | **P4 (BL6, ♂)** | **TD** | **80** | **80** | **67** | **43** |
|  |  | **FN** | 20 | 20 | 33 | 57 |
|  |  | **FD** | 0 | 0 | 0 | 0 |
|  |  | **TN** | 100 | 100 | 100 | 100 |
|  | **P5 (BALB/c, ♂)** | **TD** | **88** | **39** | **22** | **41** |
|  |  | **FN** | 12 | 61 | 78 | 59 |
|  |  | **FD** | 0 | 0 | 0 | 0 |
|  |  | **TN** | 100 | 100 | 100 | 100 |

True distressed (TD), false non-distressed (FN), false distressed (FD), True non-distressed (TN)
