# Supplementary material for: The Predictive Accuracy of Methods Commonly Used for Evaluating Animal Distress
Source: FASEB J. 2026 Jun 8;40(11):e71986. doi: 10.1096/fj.202504927RR (PMC13244802; doi:10.1096/fj.202504927RR)
Supplement: Supplementary file 10 — Table S6: Cut‐Off values based on Youden‘s index for body weight change, distress score, burrowing, and nesting behavior during pancreatitis. [file FSB2-40-e71986-s007.docx]

**Table S6:** Cut-Off values based on Youden‘s index for body weight change, distress score, burrowing, and nesting behavior during pancreatitis.

| **parameter** | **phases** | **P6 (BL6, ♂)** | | **P7 (BL6, ♂)** | | **P8 (BALB/c, ♂)** | | **P9 (BL6, ♀)** | | **P10 (BALB/c, ♀)** | |
| --- | --- | --- | --- | --- | --- | --- | --- | --- | --- | --- | --- |
|  |  | **Youden Index** | **Cut Off** | **Youden Index** | **Cut Off** | **Youden Index** | **Cut Off** | **Youden Index** | **Cut Off** | **Youden Index** | **Cut Off** |
| **Δ body weight** | **pre vs. acute phase** | no data | no data | 0.6 | 0.7 | 0.6 | 0.9 | 0.3 | 2.6 | 0.4 | -0.4 |
|  | **pre vs. early phase** | 0.6 | -1.7 | 0.7 | -1.1 | 0.6 | 0.9 | 0.6 | 0.2 | 0.6 | -0.5 |
|  | **pre vs. middle phase** | 0.8 | -1.3 | 0.7 | -2.1 | 0.6 | -1.0 | 0.6 | 1.1 | 0.5 | 0.2 |
|  | **pre vs. late phase** | 0.8 | -1.3 | 0.7 | -2.1 | 0.7 | -2.9 | 0.5 | 0.7 | 0.6 | -0.7 |
| **distress score** | **pre vs. acute phase** | no data | no data | 0.1 | 1.5 | 0.8 | 1.0 | no cut off values | no cut off values | 0.3 | 1.0 |
|  | **pre vs. early phase** | no cut off values | no cut off values | 0.1 | 1.0 | 0.9 | 1.0 | 0.1 | 1.0 | 0.3 | 1.0 |
|  | **pre vs. middle phase** | 0.2 | 1.0 | 0.5 | 1.0 | 0.8 | 1.0 | no cut off values | no cut off values | 0.5 | 1.0 |
|  | **pre vs. late phase** | 0.2 | 1.0 | 0.6 | 1.0 | 0.9 | 1.0 | 0.1 | 1.0 | 0.5 | 1.0 |
| **burrowing** | **pre vs. acute phase** | no data | no data | 0.8 | 17.0 | 0.4 | 68.0 | 0.6 | 23.0 | 0.5 | 42.5 |
|  | **pre vs. early phase** | 0.7 | 72.0 | 0.7 | 17.0 | 0.6 | 68.0 | 0.6 | 31.5 | 0.8 | 12.5 |
|  | **pre vs. middle phase** | 0.6 | 129.0 | 0.4 | 44.5 | 0.4 | 117.0 | 0.4 | 57.0 | 0.5 | 11.5 |
|  | **pre vs. late phase** | 0.6 | 71.5 | 0.2 | 60.5 | 0.5 | 42.5 | 0.3 | 62.5 | 0.3 | 47.0 |
| **nesting** | **pre vs. acute phase** | no data | no data | 0.1 | 2.0 | 0.1 | 4.5 | 0.4 | 1.5 | 0.2 | 3.5 |
|  | **pre vs. early phase** | 0.3 | 4.5 | 0.1 | 3.5 | 0.1 | 2.5 | 0.2 | 3.5 | 0.1 | 1.5 |
|  | **pre vs. middle phase** | 0.8 | 4.5 | 0.4 | 3.5 | 0.2 | 4.5 | 0.6 | 3.5 | 0.2 | 3.5 |
|  | **pre vs. late phase** | 0.7 | 4.5 | 0.3 | 3.5 | 0.1 | 2.0 | 0.6 | 3.5 | 0.3 | 3.5 |
