# Supplementary material for: The Predictive Accuracy of Methods Commonly Used for Evaluating Animal Distress
Source: FASEB J. 2026 Jun 8;40(11):e71986. doi: 10.1096/fj.202504927RR (PMC13244802; doi:10.1096/fj.202504927RR)
Supplement: Supplementary file 11 — Table S7: Overview of classification results (true/false sick/healthy) for various distress parameters during pancreatitis, when applying cut‐offs from P7 to P6, P7, P8, P9, and P10. [file FSB2-40-e71986-s011.docx]

**Table S7:** Overview of classification results (true/false sick/healthy) for various distress parameters during pancreatitis, when applying cut-offs from P7 to P6, P7, P8, P9, and P10.

| **parameter** | **project** | **status** | **acute phase** | **early phase** | **middle phase [%]** | **late phase** |
| --- | --- | --- | --- | --- | --- | --- |
|  |  |  | **[%]** | **[%]** |  | **[%]** |
| **Δ body weight** | **P6 (BL6, ♂)** | **TD** | no values | **81** | **88** | **81** |
|  |  | **FN** | no values | 19 | 12 | 19 |
|  |  | **FD** | no values | 25 | 12 | 12 |
|  |  | **TN** | no values | 75 | 88 | 88 |
|  | **P7 (BL6, ♂)** | **TD** | **88** | **81** | **73** | **73** |
|  |  | **FN** | 12 | 19 | 27 | 27 |
|  |  | **FD** | 31 | 12 | 6 | 6 |
|  |  | **TN** | 69 | 88 | 94 | 94 |
|  | **P8 (BALB/c, ♂)** | **TD** | **94** | **75** | **62** | **81** |
|  |  | **FN** | 6 | 25 | 38 | 19 |
|  |  | **FD** | 38 | 19 | 19 | 19 |
|  |  | **TN** | 62 | 81 | 81 | 81 |
|  | **P9 (BL6, ♀)** | **TD** | **31** | **38** | **33** | **47** |
|  |  | **FN** | 69 | 62 | 67 | 53 |
|  |  | **FD** | 19 | 6 | 6 | 6 |
|  |  | **TN** | 81 | 94 | 94 | 94 |
|  | **P10 (BALB/c, ♀)** | **TD** | **56** | **56** | **53** | **47** |
|  |  | **FN** | 44 | 44 | 47 | 53 |
|  |  | **FD** | 31 | 12 | 6 | 6 |
|  |  | **TN** | 69 | 88 | 94 | 94 |
| **distress score** | **P6 (BL6, ♂)** | **TD** | no values | **0** | **19** | **19** |
|  |  | **FN** | no values | 100 | 81 | 81 |
|  |  | **FD** | no values | 0 | 0 | 0 |
|  |  | **TN** | no values | 100 | 100 | 100 |
|  | **P7 (BL6, ♂)** | **TD** | **6** | **12** | **47** | **60** |
|  |  | **FN** | 94 | 88 | 53 | 40 |
|  |  | **FD** | 0 | 0 | 0 | 0 |
|  |  | **TN** | 100 | 100 | 100 | 100 |
|  | **P8 (BALB/c, ♂)** | **TD** | **81** | **88** | **81** | **94** |
|  |  | **FN** | 19 | 12 | 19 | 6 |
|  |  | **FD** | 0 | 0 | 0 | 0 |
|  |  | **TN** | 100 | 100 | 100 | 100 |
|  | **P9 (BL6, ♀)** | **TD** | **0** | **6** | **0** | **12** |
|  |  | **FN** | 100 | 94 | 100 | 88 |
|  |  | **FD** | 0 | 0 | 0 | 0 |
|  |  | **TN** | 100 | 100 | 100 | 100 |
|  | **P10 (BALB/c, ♀)** | **TD** | **31** | **25** | **53** | **53** |
|  |  | **FN** | 69 | 75 | 47 | 47 |
|  |  | **FD** | 0 | 0 | 0 | 0 |
|  |  | **TN** | 100 | 100 | 100 | 100 |
| **burrowing** | **P6 (BL6, ♂)** | **TD** | no values | **50** | **50** | **56** |
|  |  | **FN** | no values | 50 | 50 | 44 |
|  |  | **FD** | no values | 0 | 6 | 12 |
|  |  | **TN** | no values | 100 | 94 | 88 |
|  | **P7 (BL6, ♂)** | **TD** | **81** | **75** | **67** | **27** |
|  |  | **FN** | 19 | 25 | 33 | 73 |
|  |  | **FD** | 6 | 6 | 31 | 50 |
|  |  | **TN** | 94 | 94 | 69 | 50 |
|  | **P8 (BALB/c, ♂)** | **TD** | **38** | **44** | **38** | **62** |
|  |  | **FN** | 62 | 56 | 62 | 38 |
|  |  | **FD** | 12 | 12 | 12 | 19 |
|  |  | **TN** | 88 | 88 | 88 | 81 |
|  | **P9 (BL6, ♀)** | **TD** | **62** | **56** | **27** | **40** |
|  |  | **FN** | 38 | 44 | 73 | 60 |
|  |  | **FD** | 12 | 12 | 50 | 62 |
|  |  | **TN** | 88 | 88 | 50 | 38 |
|  | **P10 (BALB/c, ♀)** | **TD** | **50** | **81** | **60** | **60** |
|  |  | **FN** | 50 | 19 | 40 | 40 |
|  |  | **FD** | 12 | 12 | 25 | 44 |
|  |  | **TN** | 88 | 88 | 75 | 56 |
| **nesting** | **P6 (BL6, ♂)** | **TD** | no values | **6** | **6** | **0** |
|  |  | **FN** | no values | 94 | 94 | 100 |
|  |  | **FD** | no values | 0 | 0 | 0 |
|  |  | **TN** | no values | 100 | 100 | 100 |
|  | **P7 (BL6, ♂)** | **TD** | **0** | **31** | **0** | **7** |
|  |  | **FN** | 100 | 69 | 100 | 93 |
|  |  | **FD** | 6 | 38 | 38 | 38 |
|  |  | **TN** | 94 | 62 | 62 | 62 |
|  | **P8 (BALB/c, ♂)** | **TD** | **6** | **31** | **25** | **12** |
|  |  | **FN** | 94 | 69 | 75 | 88 |
|  |  | **FD** | 12 | 19 | 19 | 19 |
|  |  | **TN** | 88 | 81 | 81 | 81 |
|  | **P9 (BL6, ♀)** | **TD** | **0** | **69** | **27** | **27** |
|  |  | **FN** | 100 | 31 | 73 | 73 |
|  |  | **FD** | 38 | 88 | 88 | 88 |
|  |  | **TN** | 62 | 12 | 12 | 12 |
|  | **P10 (BALB/c, ♀)** | **TD** | **6** | **38** | **7** | **0** |
|  |  | **FN** | 94 | 62 | 93 | 100 |
|  |  | **FD** | 12 | 25 | 25 | 25 |
|  |  | **TN** | 88 | 75 | 75 | 75 |

True distressed (TD), false non-distressed (FN), false distressed (FD), True non-distressed (TN)
