# Supplementary material for: The Predictive Accuracy of Methods Commonly Used for Evaluating Animal Distress
Source: FASEB J. 2026 Jun 8;40(11):e71986. doi: 10.1096/fj.202504927RR (PMC13244802; doi:10.1096/fj.202504927RR)
Supplement: Supplementary file 12 — Table S8: Summary of Spearman correlations between all pairwise combinations of the four welfare markers (BW, DS, Burr, Nest) across the three disease models. For each model–pair combination, the total number of correlations tested (across projects and phases), the number reaching statistical significance, and the corresponding percentage are reported. [file FSB2-40-e71986-s003.docx]

**Table S8:** Summary of Spearman correlations between all pairwise combinations of the four welfare markers (BW, DS, Burr, Nest) across the three disease models. For each model–pair combination, the total number of correlations tested (across projects and phases), the number reaching statistical significance, and the corresponding percentage are reported.

| **Model** | **pair** | **Total No.**  **of correlations** | **Total No. of**  **Significant correlations** | **Percentage of**  **significant correlations** |
| --- | --- | --- | --- | --- |
| **Transmitter implantation** | **Burr vs Nest** | 5 | 0 | 0 |
| **Transmitter implantation** | **BW vs Burr** | 5 | 1 | 20 |
| **Transmitter implantation** | **BW vs DS** | 3 | 1 | 33 |
| **Transmitter implantation** | **BW vs Nest** | 5 | 1 | 20 |
| **Transmitter implantation** | **DS vs Burr** | 3 | 0 | 0 |
| **Transmitter implantation** | **DS vs Nest** | 3 | 1 | 33 |
| **Bile duct ligation** | **Burr vs Nest** | 5 | 1 | 20 |
| **Bile duct ligation** | **BW vs Burr** | 5 | 2 | 40 |
| **Bile duct ligation** | **BW vs DS** | 4 | 3 | 75 |
| **Bile duct ligation** | **BW vs Nest** | 5 | 2 | 40 |
| **Bile duct ligation** | **DS vs Burr** | 4 | 2 | 50 |
| **Bile duct ligation** | **DS vs Nest** | 4 | 2 | 50 |
| **Pancreatitis** | **Burr vs Nest** | 5 | 1 | 20 |
| **Pancreatitis** | **BW vs Burr** | 5 | 2 | 40 |
| **Pancreatitis** | **BW vs DS** | 4 | 3 | 75 |
| **Pancreatitis** | **BW vs Nest** | 5 | 1 | 20 |
| **Pancreatitis** | **DS vs Burr** | 4 | 1 | 25 |
| **Pancreatitis** | **DS vs Nest** | 4 | 2 | 50 |

Body weight change (BW), Distress score (DS), Burrowing activity (Burr), and Nesting behavior (Nest), number (No.).
